# Reconstructed Skin Models Revealed Unexpected Differences in Epidermal African and Caucasian Skin

**DOI:** 10.1038/s41598-019-43128-3

**Published:** 2019-05-15

**Authors:** Sarah Girardeau-Hubert, Céline Deneuville, Hervé Pageon, Kahina Abed, Charlotte Tacheau, Nükhet Cavusoglu, Mark Donovan, Dominique Bernard, Daniel Asselineau

**Affiliations:** L’Oréal Research and Innovation, 1 avenue E. Schueller, 93600 Aulnay-sous-Bois, France

**Keywords:** Skin models, Differentiation

## Abstract

Clinical observations of both normal and pathological skin have shown that there is a heterogeneity based on the skin origin type. Beside external factors, intrinsic differences in skin cells could be a central element to determine skin types. This study aimed to understand the *in vitro* behaviour of epidermal cells of African and Caucasian skin types in the context of 3D reconstructed skin. Full-thickness skin models were constructed with site matched human keratinocytes and papillary fibroblasts to investigate potential skin type related differences. We report that reconstructed skin epidermis exhibited remarkable differences regarding stratification and differentiation according to skin types, as demonstrated by histological appearance, gene expression analysed by DNA microarray and quantitative proteomic analysis. Signalling pathways and processes related to terminal differentiation and lipid/ceramide metabolism were up-regulated in epidermis constructed with keratinocytes from Caucasian skin type when compared to that of keratinocytes from African skin type. Specifically, the expression of proteins involved in the processing of filaggrins was found different between skin models. Overall, we show unexpected differences in epidermal morphogenesis and differentiation between keratinocytes of Caucasian and African skin types in *in vitro* reconstructed skin containing papillary fibroblasts that could explain the differences in ethnic related skin behaviour.

## Introduction

Ethnic skin type’s diversity in human populations reveals the multifactorial adaptation in ancestry evolution resulting in different cutaneous physiology. Differences in pigmentation, signs of ageing and stratum corneum (SC) function could reflect the versatility of epidermal functions among different skin types.

In the literature, it appears that barrier function of SC differs between skin types and has often been proposed to be greater in darkly pigmented skin (see review from Rawlings *et al*.)^1^. Nevertheless, numerous conflicting results based on measures of biophysical parameters have been noticed for barrier function. The mostly used parameters are transepidermal water loss (TEWL), conductance/capacitance, corneocytes cohesion and barrier recovery. Regarding the comparison between skins of Caucasian and African/African-American origins, some studies demonstrated higher TEWL in Caucasian skin^2–4^ unlike other studies (see review from Wesley and Maibach)^5^ or studies showing no significant variations^6,7^. For the capacitance parameters, all related to water content, a part of the published studies cited above reported a higher hydration of epidermis in African/African-American skins while others showed no differences^5,6,8^. Finally, a stronger cohesion of corneocytes or greater SC thickness in African skin has been suggested due to the number of tape strippings needed to disrupt barrier function^2,9^.

The reason for this discrepancy between published studies could be related to the measures performed on different body site or at different ages, or from environmental differences as well as the measured biophysical parameters^10^. Therefore, it appears difficult to assert that African or Caucasian skin types present a better barrier function since it depends on various factors.

Numerous studies agree that capacitance could be higher in African facial skin compared to Caucasian facial skin and may suggest a greater hydration for the former^4,11^. However, facial skin is complex since the same location could also have higher TEWL in African skin than Caucasian skin, suggesting better barrier properties for the latter^11^.

Concerning other body sites, conflicting results were observed between skin types. High prevalence of xerosis is noticed in African body skin. Indeed, African people often complain about the dry skin sensation, mainly characterised by scaly and ashy appearance of their pigmented skin and discomfort^8,12^. Compared to Caucasian skin, most of the studies have reported that African/African-American skins showed delayed overall age effects^13^. They are slightly affected in the deeper dermal component^14^ whereas the effects are more evident in the most superficial skin layers with age. Thus, skin ageing appears to alter mostly colour heterogeneity and SC barrier function in African-American skin^15^. African skin shows an increased dryness on ventral and dorsal arm similar to Caucasian skin with age but higher compared to the other skin type groups^6^.

Altogether, an identification of biological specificities in skin structure, composition and cellular function may help to understand the differential susceptibility of skin type to cutaneous disorders. Previous studies have highlighted that Dermal-Epidermal Junction (DEJ) relief and protein composition such as type IV and VII collagens differ between skin types^16,17^. We and others also noticed *in vitro* functional differences in dermal fibroblasts and keratinocytes according to their origin^18,19^. With the use of skin substitutes, Yoshida *et al*. have shown that keratinocytes from light or dark skin are involved in distinct regulation of skin pigmentation^20^. Therefore, *in vitro* full-thickness skin models are a useful tool to deepen our understanding of the skin type’s biology, as it takes into account the 3D architecture and the use of isolated primary cells, dermal fibroblasts and keratinocytes, from the same donor. Previous studies from our laboratory have shown the potential of reconstructed skin (RS) models to reveal skin differences which had not been seen previously in *in vivo* studies^21,22^.

The native epidermal barrier function critically depends on a correct terminal differentiation and SC formation and composition. It has been shown that processing of filaggrin and lipid metabolism were key determinants in epidermal functions^23^. These processes lead to natural moisturizing factor (NMF) production and ceramide composition, respectively, elements of the outermost layers of the SC, key to skin barrier function^24,25^. The purpose of this study was to analyse the *in vitro* biological epidermal processes related to terminal differentiation occurring in African and Caucasian reconstructed skins, independent of the pigmentation processes. In this report, the capacity of keratinocytes to form a fully differentiated and keratinised epidermis on a fibroblast-populated dermis was evaluated according to their origin. A wide exploration of mRNA and protein expression levels in the epidermis of reconstructed skin was undertaken to elucidate the differential *in vitro* functions of keratinocytes of African and Caucasian origins.

## Results

### *In vivo* differences in the proliferation and differentiation state of epidermis between African and Caucasian skins

**Figure 1:**
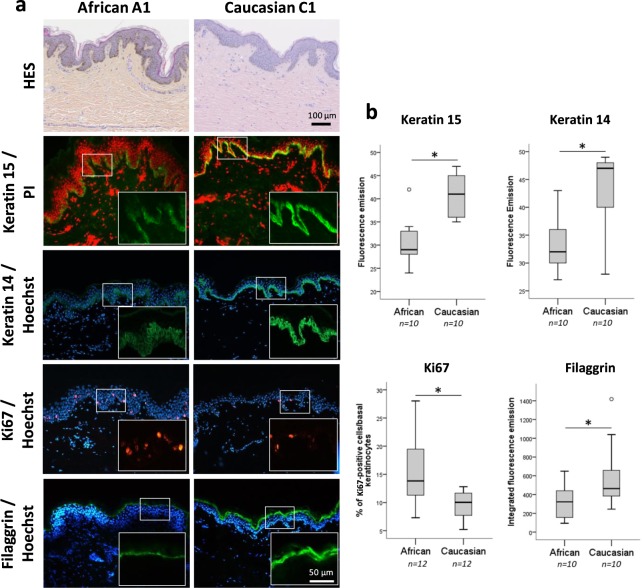
Differential proliferating/differentiation state of epidermis between African and Caucasian human skin types. (**a**) Sections of human skin stained with HES and keratin 15, keratin 14, Ki67 and filaggrin immunofluorescence detections in epidermis for donors A1 and C1. Nuclei were counterstained with propidium iodide PI (red) or Hoechst (blue). Scale bars: 100 µm for HES and 50 µm for immunostainings (insets: 25 µm). Note that keratin 15 staining was pronounced in the deeper part of epidermal rete ridges in African skin whereas it was detected throughout the basal layer of epidermis in Caucasian skin. (**b**) Quantification of fluorescent detection for keratin 15, keratin 14 and filaggrin and amount of Ki67 positive cell in basal layer of epidermis in African and Caucasian skin (*n* = 10–12 donors per skin type). Significant difference between African and Caucasian RS group was determined using the two-tailed unpaired Student’s t-test. *P ≤ 0.05.

African human skin is characterised by a high rate of convolution of the DEJ when compared to Caucasian skin (Fig. 1a). Keratin 15 (K15), which has often been associated with slow-cycling cells and co-expressed with the K5/K14 pair, was restricted to the basal layer of epidermis in all samples. In Caucasian epidermis, K15 positive cells were homogeneously distributed along the basal layer whereas in African epidermis, they were mostly found in the deepest part of the epidermal rete ridges (Fig. 1a). Stronger staining of K15, as well as K14, was observed in Caucasian skin (Fig. 1b). We also investigated the proliferating state of basal keratinocytes through the detection of the Ki67 marker. The number of positive cells indicated more abundant proliferating cells in native epidermis of African skin when compared to Caucasian skin. The epidermis from African and Caucasian normal human skin appeared histologically similar in terms of differentiation. Nevertheless, numerous African donors showed lower immunofluorescence levels for filaggrin in the terminal differentiated epidermis layers when compared with Caucasian donors, as observed for donors A1 *vs* C1 (Fig. 1a,b).

Overall, distinct epidermal markers were identified suggesting that keratinocyte populations were heterogeneous between African and Caucasian skin types. In particular, we noticed that African epidermis displayed more dividing cells but less terminal differentiation as suggested by the filaggrin detection. Thus, we hypothesise that it could reflect different epidermal programs in each skin type, in particular at the level of differentiation, and this may be highlighted through analyses of *in vitro* 3D reconstruction skin models using cells from both skin types.

### Distinct transcriptomic profile of epidermis from reconstructed skin models according to skin type

**Figure 2:**
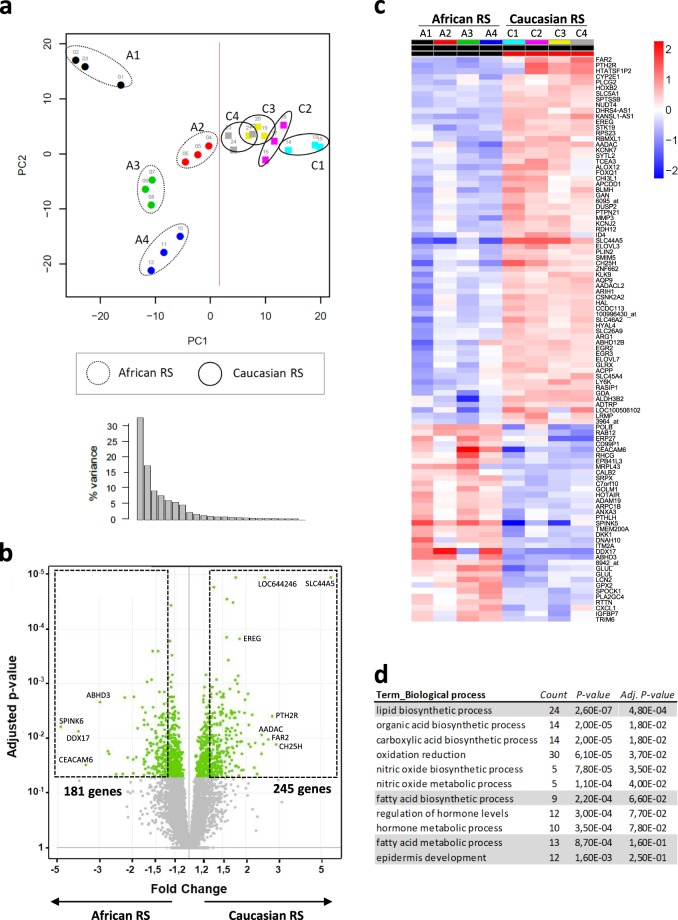
Gene expression analysis of epidermis from RS reveals specific profile according to the origin of skin cells used in the *in vitro* model. (**a**) Principle component analysis of sample expression intensities using whole gene datasets from epidermis of African and Caucasian RS (*n* = 4 donors per skin type in triplicate experiments). (**b**) Volcano plot of differentially expressed genes between epidermis of Caucasian and African RS from a list of 426 probesets with Fc ≥ 1.3 and FDR adjusted *P* ≤ 0.05. (**c**) Heatmap of top 100 significant differential genes from the 426 regulated probesets between epidermis of African and Caucasian RS. On the X axis, each column indicates a replicate sample and they are grouped per donor and per skin type. (**d**) Gene ontology classification of most significant biological processes reflecting differences between epidermis from African and Caucasian RS (*P*-value and FDR adjusted *P*-value).

Considering the observations in histological and biomarkers immunostainings on skin samples, we have chosen to reconstruct skin with cells from 4 donors of African skin types and 4 donors of Caucasian skin types selected with similar age range. We also chose to perform a large omics analysis of the *in vitro* epidermis at 7–9 days of the emersion culture phase, which is the critical time for complete *in vitro* differentiation. Indeed, gene expression analysis of keratinocytes revealed that epidermis from African RS and Caucasian RS were characterised by different profiles. Unsupervised analyses were performed using the signals of all the probes spotted on the microarray. Firstly, since the *in vitro* skin model is a critical step-by-step culture reconstruction that could lead to additional biological bias, it was very important to observe that replicate profiles of RS for each donor were similar. In Fig. 2a, the principle component analysis showed the triplicate samples of each donor well grouped together, indicating a good reproducibility of gene expression between RS models within the epidermis of each donor. The two first principle components (PC) revealed the highest variances between samples, 33% for the PC1 and 18% for the PC2, respectively. The PC1 axis segregated the RS samples according to their skin type origin: it indicates that the probe sets that support this axis are a part of the signature differentiating the epidermis of the RS of the two origins.

Secondly, supervised analyses were performed with the statistical model applied to identify the differentially expressed genes. The volcanoplot on the Fig. 2b illustrates the 426 significant probe sets differentially expressed based on adjusted *P* value < 0.05 and absolute fold change (|Fc|) ≥ 1.3 between epidermis from African RS and Caucasian RS: 181 epidermal expressed genes were up-regulated in African epidermis and 245 were up-regulated in Caucasian epidermis (Supplementary Table S1). The clustering of the top 100 genes was represented with the Heatmap (Fig. 2c). Some of them were particularly up-regulated (|Fc| > 2) between the African and Caucasian skin models (10 and 16, respectively) including *SPINK6* (serine peptidase inhibitor, Kazal type 6)*, DDX17* (DEAD-box helicase 17), *CEACAM6* (carcinoembryonic antigen related cell adhesion molecule) for African RS and *SLC44A5* (solute carrier family 44 member 5*), CH25H* (cholesterol 25-hydroxylase), *PTH2R* (parathyroid hormone 2 receptor) for Caucasian RS. The top 100 genes also revealed that some of them were related to epidermal differentiation processes.

Considering the 426 differentially regulated probe sets, several lipid metabolism processes (GO:0008610, GO:0006633, GO:0006631) and epidermis development process (GO: 0008544) were identified to be either significantly changed or a trend (adjusted P value < 0.05) in the top GO classification for biological processes (Fig. 2d). Analysis of expressed genes linked to lipid metabolism suggested that they were linked to epidermal formation of long-chain ceramides (Supplementary Table S2a) and those linked to filaggrin processing led to increased terminal differentiation in Caucasian RS epidermis (see Supplementary Table S2b).

**Figure 3:**
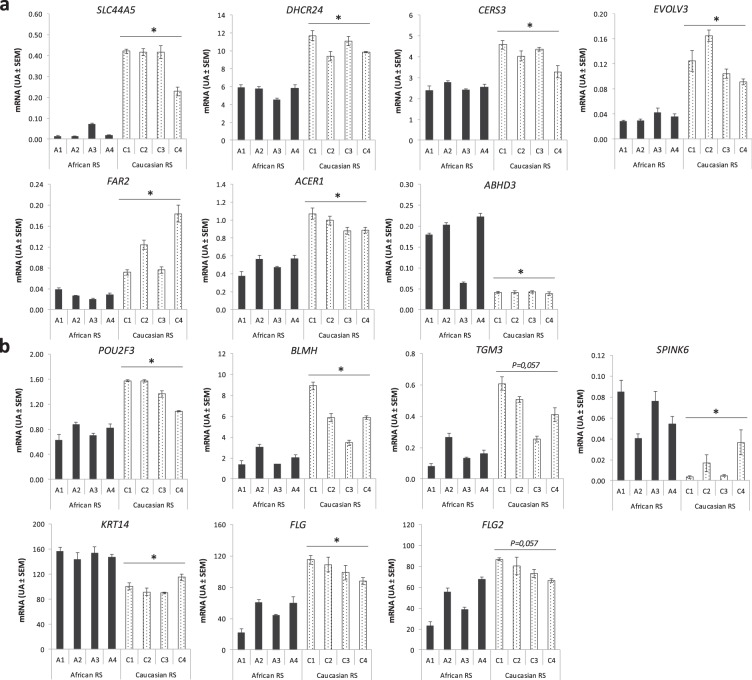
The differential expression of African and Caucasian epidermal signature genes in keratinocytes of reconstructed skins. The relative quantification of mRNA was performed by RT-qPCR for lipid metabolism-related genes: *SLC44A5, DHCR24, CERS3, EVOLV3, FAR2, ACER1, ABHD3* (**a**) and differentiation processes-related genes: *POU2F3, BLMH, TGM3, SPINK6, KRT14, FLG* and *FLG2* (**b**). Mean are reported ± SE (*n* = 3 replicates per donor). Statistical significance, denoted with * (*P* < 0.05), between African and Caucasian RS groups (*n* = *4* donors per skin type) are determined using the non-parametric Mann-Whitney test.

mRNA quantification by RT-qPCR of 10 genes identified in transcriptomic analyses confirmed to be differentially regulated in epidermis from African and Caucasian RS: *SLC44A5*, *DHCR24*, *CERS3*, *EVOLV3*, *FAR2*, *ACER1*, *ABHD3* (chosen for lipid metabolism) and *POU2F3*, *BLMH*, *SPINK6* (chosen for differentiation processes) (Fig. 3). *KRT14*, *FLG*, *FLG2* were also analysed and were differentially regulated between the models. Interestingly, the most up-regulated epidermal gene (mean Fc 12.5) in Caucasian skin models was *SLC44A5*, coding for a choline transporter. This gene was already identified in a previously published transcriptomic study on African, Caucasian and Asian skins^26^. By contrast, *SPINK6*, a protease inhibitor, was the highest up-regulated gene in African RS models (mean Fc -4.2). The active form of SPINK6 inhibits the family of kallikrein-related peptidases involved in the cleavage of corneodesmosomes in the epidermal desquamation processes^27^. CEACAM6, involved in cell adhesion and up-regulated gene in three African RS models (mean Fc -4,6), was another interesting marker overexpressed in psoriasis skin and linked to a de-differentiated state of keratinocytes^28^. Overall, these results indicate a higher level of differentiated epidermis in Caucasian RS as compared to African RS.

### Distinct epidermal differentiation pattern in African and Caucasian skin models are in part related to filaggrin processing mechanisms

**Figure 4:**
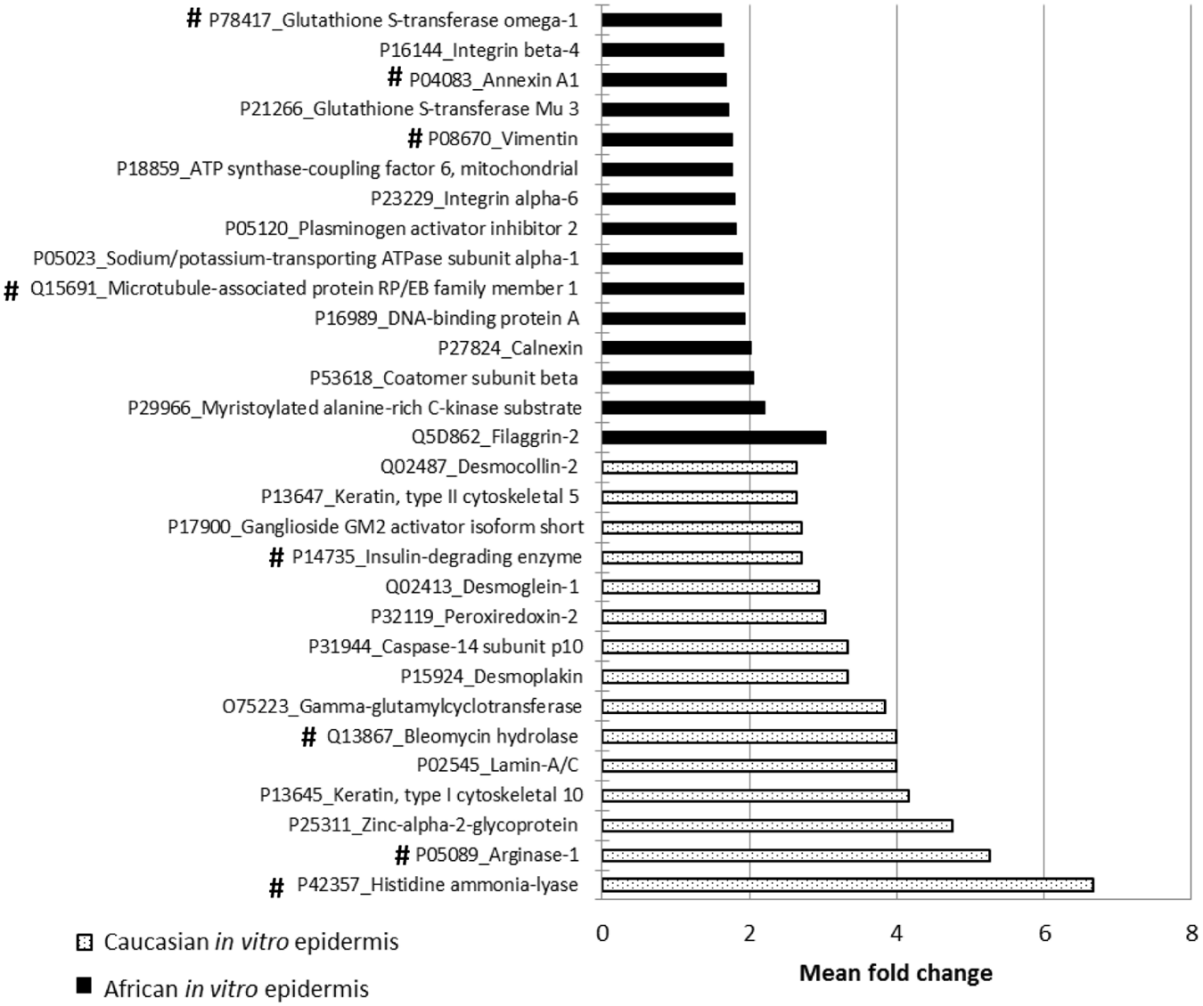
Regulation of proteins related to differentiation processes discriminates epidermis of African and Caucasian RS. Top 15 of differentially regulated proteins in epidermis from African and Caucasian *in vitro* RS. Experiments producing RS model with cells from donors A1 and C2 were performed in triplicate. Identified proteins from RS epidermis by at least 2 peptides or more in proteomic analyses were considered differentially produced with *P* ≤ 0.05 and |Fc| ≥ 1.2 and proteins were labelled in graph by the accession number and the official full name. Note that a part of the regulated proteins is related to the processing of filaggrin such as bleomycin hydrolase, histidine ammonia-lyase, arginase and caspase 14. # indicated regulations observed in the transcriptomic analysis in Fig. 2.

We next examined whether the transcriptome differences reflect change in the proteome for epidermal differentiation between the *in vitro* models. SILAC-based quantitative proteomics was performed to identify changes between the epidermis from an African RS (donor A1) and a Caucasian RS (donor C2) at day 9 of emersion phase. Up to 1995 proteins (with at least 2 peptides) were identified by Mascot with 1% FDR (Fig. 4). 98 proteins were up-regulated in Caucasian RS and 34 proteins from African RS (*P* ≤ 0.05 and Fc ≥ 1.2). For the top 15 up-regulated proteins in African epidermis and Caucasian epidermis from RS, some of them were also up-regulated in the transcriptomic analysis (Fig. 4, labelled with #) suggesting that a higher level of differentiation could occur in epidermis of Caucasian RS.

Indeed, gene expression of histidine ammonia-lyase (HAL), arginase 1 (ARG-1), insulin degrading enzyme (IDE) and bleomycin hydrolase (BLMH) were up-regulated at the protein level in *in vitro* epidermis from Caucasian RS performed with cells from 4 donors. Most of these proteins and other newly identified enzymes, like such as gamma-glutamylcyclotransferase (*GGCT* gene) and caspase-14, supported the higher level of SC maturation in Caucasian RS. Additionally, K10, desmoplakin, desmoglein-1, desmocollin-2, proteins involved in the corneodesmosomal complex and the differentiation of the keratinocytes, were also expressed at a higher level in Caucasian RS.

By contrast, in the African *in vitro* epidermis, proteins related to the basal layer like α6/β4 integrins were increased which could indicate the maintenance of keratinocyte basal layer properties. Surprisingly, filaggrin 2 was the most up-regulated identified protein in the African epidermal RS while the transcriptomic study indicated that *FLG* gene was decreased in African RS and no change could be seen in *FLG2* gene. Filaggrin 2 is a fused S100 domain protein that shares common structural features with filaggrin^29^. The hydrolysis of filaggrin is one of the major processes leading to NMF production in SC. However, in some circumstances, it has been reported that minor changes in filaggrin degradation led to accumulation of high molecular weight peptides^30^. In this study, identified peptides covered 10% of filaggrin 2 sequence with 2 peptides from the N-terminal region, 1 peptide from the first repetitive region A and others from the second repetitive region B (Supplementary Fig. S1a). Therefore, this observation may suggest that filaggrin 2 is less processed in epidermis of African RS than those of Caucasian RS. In order to observe the localization of filaggrin 2 in the *in vitro* differentiated epidermis, two antibodies for filaggrin 2 were used for the detection of the Ser-rich region and the N-terminal domain. Firstly, immunodetection of the filaggrin 2 Ser-rich region suggested that filaggrin 2 was unusually retained in the spinous and the granular epidermal layers of African RS whereas it was mostly found in the last granular epidermal layers and the accumulated SC of Caucasian RS in *in vitro* conditions (Supplementary Fig. S1b). The detection of N-terminal domain for filaggrin 2 also revealed its synthesis in epidermis of RS. A higher detection was observed both in granular epidermal layers and SC of Caucasian RS when compared to African RS. Accumulation of the N-domain in SC may highlight both a higher level of synthesis and processing of filaggrin 2 in epidermis of Caucasian RS. Therefore, the proteomic result for filaggrin 2 in African RS appeared to reflect the quantification in the living epidermis. Instead, filaggrin 2 was mostly found in the SC, a sign of a higher processing in Caucasian epidermis compared to African epidermis in *in vitro* conditions of RS.

**Figure 5:**
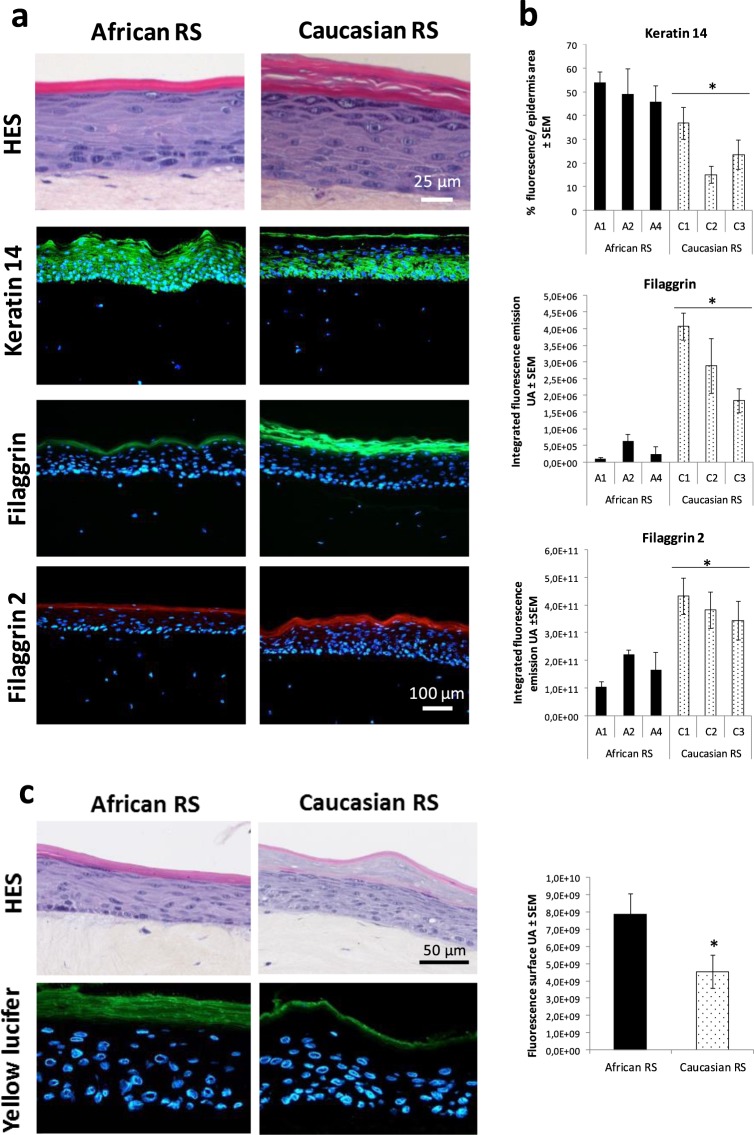
Higher differentiation processes of keratinocytes in epidermis of Caucasian *in vitro* RS models compared to those in epidermis of African RS. (**a**) Representative histology of RS and protein expression patterns in the different epidermal layers for keratin 14, filaggrin and filaggrin 2 in African and Caucasian RS. Nuclei were counterstained with DAPI. Scale bars: 25 µm for HES and 100 µm for immunostainings. Note that epidermis of Caucasian RS showed more granular layers, indicated by arrows, as compared to that of African RS (**b**) Quantification of immunofluorescent detection of keratin 14, filaggrin and filaggrin 2 (N-term region) in RS (*n* = *3* donors per skin type). Mean are reported ± SE (*n* = 3 replicates per donor). (**c**) Permeability to Lucifer yellow was tested at day 9 of the emersion phase of new RS experiments. Mean are reported ± SE (*n* = *4* donors per skin type). Note that fluorescent detection showed that the dye was retained in the SC of epidermis of both African RS and Caucasian RS. However, it appears that fluorescent dye have diffused down more in the SC layers of African RS. Scale bar: 50 µm. Statistical significance between African and Caucasian RS groups were determined using the non-parametric Mann-Whitney test, denoted with *(*P* < 0.05).

Next, new cultures of RS were performed with 3 donors per group to analyse the expression pattern of protein set mostly related to terminal differentiation at day 8 of the emersion phase. Histological sections underlined that epidermis from Caucasian RS showed more granular layers characterized by the keratohyalin granules as compared to those from African RS (Fig. 5a). The observations also indicated a high, gradual accumulation of corneocytes layers with limited compaction of SC in the epidermis of Caucasian RS but without desquamation, an aberrant phenomenon typically observed in the *in vitro* epidermis. On one hand, parakeratosis was observed in the epidermis from African RS. In contrast to *ex vivo* skin, *in vitro* epidermis of African RS displayed abnormal persistent detection of K14 in all epidermal layers suggesting that *in vitro* differentiation was impaired in keratinocytes from African skin type (Fig. 5). On the other hand, epidermis from Caucasian RS showed a marked expression of filaggrin 1 and 2 in the terminal layers (Fig. 5a,b). No significant differences were observed in involucrin and loricrin distribution (Supplementary Fig. S2) supporting that RS models were different for filaggrins at this specific time point of cultures and terminal epidermal differentiation was higher in Caucasian RS than in African RS.

We hypothesise that changes in epidermal differentiation and ceramide composition could alter epidermis permeability. Percutaneous penetration occurs through paracellular and intracellular pathways and through appendages. A Lucifer yellow assay was used to test the outside-in permeability of SC in these RS models. The hydrophilic fluorescent dye Lucifer Yellow permeability assay, usually used when the magnitude of epidermal permeability barrier disruption is large, showed no considerable differences in diffusion of Lucifer yellow in African and Caucasian epidermis from RS. In both African and Caucasian models, the hydrophilic dye was retained in the SC and no diffusion was observed in the living epidermis or into the dermal layer (Fig. 5c) suggesting that epidermal barrier was effective for this molecule. Nevertheless, higher diffused staining was seen in the SC of African models in comparison to Caucasian models reflecting a higher penetration of dye in the SC of the former. Thus, this suggested that the cells of different skin types lead to moderate changes in permeability of RS epidermal terminal layers. These results showed that the less differentiated African *in vitro* epidermis corresponded to increased permeability of the SC suggesting a correlation between morphological and functional data.

## Discussion

In this report, we highlighted that epidermal differentiation of the *in vitro* RS appeared strongly influenced by the skin type origin of the cells used. Caucasian keratinocytes underwent an improved terminal differentiation *in vitro* when compared to African keratinocytes in RS, as demonstrated by the histologic, transcriptomic and proteomic results.

During *in vivo* differentiation, the levels of phospholipids are progressively reduced and replaced by free fatty acids, glycosphingolipids and ceramides. This provides a hydrophobic extracellular environment in the outermost layers of the SC, mainly composed of long-chained ceramides (50%), saturated fatty acids (25%) and cholesterol (15%)^31^. In this report, lipid metabolism-related genes were enriched in the gene ontology classification between the African and Caucasian *in vitro* epidermis suggesting that numerous classes of lipids were modulated^32^. Indeed, the first biological process from the gene ontology analysis was a lipid biosynthetic process represented by 24 genes. The most up-regulated gene observed in *in vitro* Caucasian epidermis, *SLC44A5*, has been identified in a transcriptomic study of the native epidermis of Caucasian ancestry when compared to African^26^. In this study, the authors have underlined *SLC44A5* as an interesting marker which is related to pigmentation phenotype of the mouse eye but little is known about the different functions it could have. SLC44A5 is a choline transporter and choline is a component of the major phospholipids of cell membranes^33^. SLC44A5 could increase choline efflux, initially released from the metabolism of sphingomyelin and phosphatidylcholine, and may also be an essential component of the lipid metabolism. Interestingly, the identification of lipids metabolism-related genes showed that ceramide processing appeared to be the most discriminating marker. Regulation of enzyme mRNAs involved in the different phase of lipid metabolism was detected, thus the modulation of the pathway is an early event in epidermis between African and Caucasian models. Interestingly, this *in vitro* specificity could be related to published reports that have shown that ceramide amount was lower in the SC from normal African epidermis as compared to Caucasian epidermis^2,34,35^.

The results also suggest that regulation of ceramide processing could explain, in part, the observed morphologic differences in differentiation between African and Caucasian epidermis from RS. In UGCG-deficient mice, the keratinocyte differentiation appeared to be altered; genes involved in lipid signalling were differentially regulated concomitant to the epidermal differentiation signalling disturbing the formation of the cornified envelope^36^. Some other studies have shown that ceramides or their derivatives can induce apoptosis and keratinocyte differentiation^37^. In our study, the epidermis of Caucasian RS presented an up-regulated signalling for ceramide metabolism and an up-regulated set of genes associated with late epidermal differentiation in contrast to those from African RS epidermis; most of them were related to profilaggrin synthesis and processing.

Filaggrin processing leads to the production of NMF mainly composed of free amino acids and their derivatives which also include lactic acid, urea, citrate, and sugars^25^. A correlation between NMF production and hydration of epidermis has been previously reported, suggesting that it serves as providing natural humectants of the outermost layers of the SC^38^. It has been reported that abnormal filaggrin processing impaired skin barrier function. Mutations associated with loss-of-function within the *FLG* genes have been identified in some cutaneous disorders such as *Ichthyosis vulgaris* characterized by dry, scaly skin^39^ and atopic dermatitis^40,41^. Some studies showed that the knockdown of filaggrin in epidermis altered barrier function and keratinocyte differentiation at the mRNA and protein level for other proteins of differentiation^42,43^ while others did not notice any alteration in a full-thickness skin equivalent^44^.

In this report, no differences could be seen at the histological level between African and Caucasian human epidermis, since stratum granulosum is highly compact in *ex vivo* skin. However, filaggrin was detected at a lower level in most African epidermis when compared to Caucasian epidermis. In the *in vitro* RS models, it was especially observed that filaggrin was more expressed and processed in Caucasian epidermis than African epidermis of RS. Profilaggrin, synthesized in granular cells, is gradually processed to free amino acids such as histidine, glutamine and arginine through the action of multiple enzymes^45^. Caspase-14 and bleomycin hydrolase are initially required for the breakdown of deiminated filaggrin into amino acids^46^. GGCT is involved in the formation of pyroglutamic acid or pyrolidine carboxylic acid (PCA) from glutamine; HAL produces endogenous urocanic acid (UCA) from the histidine and ARG-1, l-ornithine and urea from the arginine^47,48^. Most enzyme genes were differentially modulated suggesting that the production of NMF could be different between African and Caucasian *in vitro* RS. Overall, the difference in profilaggrin deposition and its processing as well as ceramide composition may change the barrier function of SC.

In the skin, the DEJ tightly binds the epidermis to the papillary dermis, determines the attachment and the polarity of basal keratinocytes and is a selective barrier for all kinds of exchanges between the two compartments^49^. In our previous work, we have identified differences between African and Caucasian human skin types specifically related to the papillary dermis^18^. Differential relief of epidermal dermal invaginations, composition of extracellular matrix at the DEJ and secretory profile from fibroblasts in monolayer indicated that papillary dermis could imply a distinct regulation of the overlying epidermal morphogenesis for the different skin types^14,16–18^. Future studies will be important to understand how the papillary fibroblasts contribute to the epidermal phenotypes observed in the *in vitro* African and Caucasian skin models.

We considered that reconstructed skins made with primary cells could be a useful way to identify specific epidermal functions as these take into account three-dimension differentiation as well as dermal-epidermal interactions. The epidermis from *in vitro* RS mostly reflects the differentiated state of *in vivo* epidermis with well characterised layers making the RS model a very useful tool to study the process of stratification and differentiation in a short period of time. However, we need to take into account that one of the limits of the *in vitro* culture of the epidermis is that SC barrier function is impaired due to an incomplete desquamation process and the amount, composition and organisation of lipids in these models are quite different from *in vivo* skin^24,50^. Additionally, beside keratinocytes and fibroblasts, we considered that our *in vitro* system could gain in functionality if other cell types such as immune cells could be included and investigations extended to inflammatory pathways. Previous studies showed that melanocytes also contributed to the enhancement of the barrier function^51,52^. Melanosome secretion participated in the acidification of epidermis and could influence the activity of numerous enzymes in the SC, which are dependent on pH^51^. In this report, the analyses of RS have shown, for the first time, new *in vitro* features of African and Caucasian skin types. Therefore, it will be of interest to consolidate these observations with additional donors with a similar age range and to open up new areas of research with Asian reconstructed skin models.

In conclusion, the present study underlined that it is essential to take into account the origin of cells in the elaboration of the 3D *in vitro* skin biological models. The unexpected differences in the *in vitro* behaviour of keratinocytes on fibroblasts-populated dermis according to cell origin suggested that they could contribute in part to the specific physiology between skin types and could be crucial in a strategy to study and adapt the cosmetic products to the different skin types.

## Methods

### Skin samples and cell isolation

Normal human skin was obtained from surgical residues of breast reduction surgery in France, with the patients’ written informed consent in accordance with the Helsinki Declaration and with Article L. 1243–4 of the French Public Health Code. Patients’ written informed consents were collected and kept by the surgeon. The samples were anonymised before their reception by the authors. Only age, sex and anatomical site of samples were specified to the authors. The authors did not participate in sample collection. Given its special nature, surgical residue is subject to specific legislation included in the French Code of Public Health (anonymity, gratuity, sanitary/safety rules and no publicity for donation). This legislation does not require prior authorisation by an ethics committee for sampling or use of surgical waste. The European Caucasian skin types and African skin types were classified as phototypes II/III/IV and phototypes V/VI, respectively according to macroscopic and melanin observations on Fontana-Masson staining (18–42 years-old; mean age 28 years ±7; n = 12 per skin phototype; Supplementary Table S3) and stated as Caucasian and African in this report.

Skin samples for cell isolation were selected according to quality and histological criteria. A large size (25 cm^2^) and the preservation of dermis were required for the cell isolation. Considering the histological quality criteria and expression of biomarkers, the keratinocytes and papillary dermal fibroblasts were isolated from the skin biopsies of 4 donors from African skin types and 4 donors from Caucasian skin types with similar age range (mean age 24 years ±2) for the following *in vitro* experiments. Briefly, fibroblasts were isolated from the superficial layer of dermis of skin biopsies with the use of a dermatome (Micro-Line Dermatome Aesculap, B.Braun Medical SAS, Boulogne, France) as previously described^53^ and used at passage 4 for reconstructed skins. Keratinocytes were obtained and cultured on a feeder layer of Swiss 3T3 fibroblasts and used at the first passage for reconstructed skins. All cells were grown at 37 °C in a humid atmosphere containing 5% CO_2_ and culture medium was changed three times per week^53^.

### Reconstructed skins

Skin equivalents were made in triplicate as previously described^54,55^. Due to the complexity of the reconstruction, three or four donors per skin type group (under 30 years-old) have been used for *in vitro* cultures and, keratinocytes and papillary fibroblasts from the same donor were matched for the reconstruction of the *in vitro* skin. Dermal equivalents were prepared with 1 × 10^6^ dermal fibroblasts and a solution of bovine type I collagen with a final concentration at 1.5 mg/ml (Collagen, Symatèse, Lyon, France). The gel solution was kept in culture for 4 days resulting in contraction of the lattice. At the top of the lattice, keratinocytes were seeded and lattices were kept in culture for 7–9 days with MEM supplemented with NEA, L-Glutamine (2 mM), sodium pyruvate, penicillin-streptomycin (30 U/µg/ml), amphotericin B (25 ng/ml), EGF (10 ng/ml), cholera toxin (0.1 nM), hydrocortisone (0.4 µg/ml) and 10% FCS. The cultures were then raised to the air-liquid interface (emersion phase) during 7–9 days to obtain a complete differentiated epidermis. Medium was changed three times per week^53^.

### Gene expression analysis using microarrays

Reconstruction of skin models was made with keratinocytes and fibroblasts from four different donors per skin type and experiments were conducted in triplicate for each donor. At the end of culture, the epidermis from reconstructed skin was manually separated from the dermal equivalent part in order to analyse gene expression in keratinocytes only. The total RNA of epidermis *vitro* samples was obtained according to the manufacturer’s instructions using Rneasy mini-kit (Qiagen, Hilden, Germany). Then, RNA samples were labelled with biotin and hybridation was performed on Affymetrix Human Genome U133 + PM Array Plates. Sample processing was performed by the Affymetrix Service Provider “KFB - Center of Excellence for Fluorescent Bioanalytics” (Regensburg, Germany; www.kfb-regensburg.de).

Microarray data analyses were performed by AltraBio (Lyon, France; www.altrabio.com). Raw data were treated with the RMA algorithm^56^. The differentially expressed genes between the two skin types were identified using a mixed linear regression model with a random ‘Donor’ effect to account for the hierarchical nature of the experimental design^57^ and the empirical Bayes method was used to compute moderated *P*-values that were then corrected for multiple comparisons using the Benjamini and Hochberg’s false discovery rate (FDR) controlling procedure^58^. Genes presenting a fold change value |FC | ≥ 1.3 and a FDR adjusted *P* value < 0.05 were selected as differentially expressed.

Gene set enrichment analyses based on the hypergeometric distribution applied to the Gene Ontology^59^ and review of the PubMed database were performed to characterize the genes differentially expressed. See more details in Supplementary Methods.

### Quantitative real-time reverse transcriptase–PCR

This study benefited from the facilities and expertise of the QPCR platform of IMAGIF (Centre de Recherche de Gif – www.imagif.cnrs.fr, France). Purified RNA quality was analysed using the Bioanalyser 2100 (Agilent Techn., Palo Alto, CA, USA) and quantified using the Nanodrop spectrophotometer (Thermo scientific., Waltham MA, USA). RNA was reverse transcribed into cDNA using High Capacity cDNA Reverse Transcription Kit (Applied Biosystems/ThermoFischer Sc.).

TaqMan experiments were carried out on an QuantStudio 12 K Flex detection system using Taqman fast advanced master mix and Taqman gene expression assays (ThermoFisher Sc.) according to the manufacturer’s recommendations (probes in Supplementary Table S4) for human *SLC44A5, DHCR24, CERS3, EVOLV3, FAR2, ACER1, ABHD3, POU2F3, BLMH, TGM3, SPINK6, KRT14, FLG, FLG2* and for the expression of three housekeeping genes (*GUSB, TBP, YWHAZ*) selected with GeNorm and Normfinder functions in the Genex software (MultiD, Göteborg, Sweden). The expression of each target gene for each sample, normalized with the average of the expression of the 3 housekeeping genes, were calculated with the Ct method^60^.

Data were statistically analysed using the non-parametric unpaired Kruskal-Wallis ANOVA test to compare the effect of different donor for each skin type group. As technical replicates were homogeneous, donors were considered as unique samples and the Mann-Whitney test was applied to compare the effect of skin type between African and Caucasian samples. *P* value < 0.05 was considered as significant.

### Quantitative proteomic analysis

Stable isotope labelling by amino acids in cell culture (SILAC) was performed to detect differences in newly synthesized proteins from epidermis between African and Caucasian skin models (donor A1 *vs* donor C2) in triplicate experiments. Proteins from Caucasian (labelled with heavy isotopes) and African RS (light, normal) were subjected to mass spectrometry experiments. Proteins identified by 2 peptides or more and with 1% false discovery rate (FDR) were selected and considered differentially produced between epidermis of African and Caucasian skin models with a *P* value < 0.05. See more details on Supplementary Methods.

### Histology and immunohistochemistry

Samples of human skin and RS were fixed in neutral formalin and paraffin sections were made and stained with haematoxylin, eosin and saffron (HES coloration). Immunostainings were performed on air-dried cryosections (5 µm). Keratin 15 and 14 were detected by monoclonal anti-human antibodies (clone LHK15, Labs Biotechnology Inc., London ON, Canada and clone RCK107, Progen, Heildeberg, Germany, respectively). Analysis of cell proliferation was performed with immunostaining of Ki-67 marker (antibody clone MM1; Novocastra, Leica Biosystems, Newcastle Ltd, UK). Filaggrin 2 was recognized with an AlexaFluor 546-conjugated monoclonal antibody fragment against the N-terminal region (Biorad, Puchheim, Germany) and with a polyclonal antibody against the SER rich region (Abcam, Cambridge, UK). Filaggrin/profilaggrin was detected with a mouse anti-human antibody (clone AKH1 from Santa Cruz techn, Dallas, TX, USA). Primary antibodies were recognised by antibodies coupled with an AlexaFluor 488 (Molecular Probes/ThermoFischer Sc). Nuclei were counterstained using propidium iodide or Hoechst (Sigma-Aldrich, Saint-Louis, MO, USA) or DAPI contained in mounting medium (SouthernBiotech, Birmingham, AL, USA).

Immunolocalization intensity analysis was performed using the image analysis software Histolab (Microvision instrument, Evry, France). Relative protein expression was quantified by fluorescence signal and/or area positively stained for each picture of tissue section. *In vivo* data was analysed using Shapiro-Wilk normality test and the two-tailed unpaired Student’s t-test. *In vitro* data was analysed using the non-parametric unpaired Kruskal-Wallis ANOVA and Mann-Whitney tests to compare the effect of donors and skin type. A *P* value < 0.05 was considered as significant.

## Supplementary information

Supplementary data and methods information
