# Author Correction: Reconstructed Skin Models Revealed Unexpected Differences in Epidermal African and Caucasian Skin

**DOI:** 10.1038/s41598-020-74970-5

**Published:** 2020-10-21

**Authors:** Sarah Girardeau-Hubert, Céline Deneuville, Hervé Pageon, Kahina Abed, Charlotte Tacheau, Nükhet Cavusoglu, Mark Donovan, Dominique Bernard, Daniel Asselineau

**Affiliations:** L’Oréal Research and Innovation, 1 avenue E. Schueller, 93600 Aulnay-sous-Bois, France

Correction to: *Scientific Reports* 10.1038/s41598-019-43128-3, published online 15 May 2019

The link to the original data is incomplete. The following section text should be included:

“Data availability

The transcriptomics data has been deposited in the ArrayExpress database at EMBL-EBI (https://www.ebi.ac.uk/arrayexpress) under accession number E-MTAB-9433.

The proteomics data has been deposited in the MassIVE database (https://massive.ucsd.edu/ProteoSAFe/static/massive.jsp) under the identifier MSV000086147.”

