# Supplementary material for: Reconstructed Skin Models Revealed Unexpected Differences in Epidermal African and Caucasian Skin
Source: Sci Rep. 2019 May 15;9:7456. doi: 10.1038/s41598-019-43128-3 (PMC6520399; doi:10.1038/s41598-019-43128-3)
Supplement: Supplementary file 1 — Supplementary data and methods information [file 41598_2019_43128_MOESM1_ESM.pdf]

RECONSTRUCTED SKIN MODELS REVEALED UNEXPECTED DIFFERENCES IN EPIDERMAL AFRICAN AND CAUCASIAN SKIN

Sarah GIRARDEAU-HUBERT, Céline DENEUVILLE, Hervé PAGEON, Kahina ABED, Charlotte TACHEAU, Nükhet CAVUSOGLU, Mark DONOVAN, Dominique BERNARD and Daniel ASSELINEAU

Supplementary Information

a

Official symbol	Official full name	EntrezID	Pseudo Fc	Adjusted p-value
SLC44A5	solute carrier family 44, member 5	204962	5,7	1,1E-05
CH25H	cholesterol 25-hydroxylase	9023	2,9	1,3E-02
PTH2R	parathyroid hormone 2 receptor	5746	2,8	4,0E-03
FAR2	fatty acyl CoA reductase 2	55711	2,6	1,1E-02
KANSL1-AS1	KANSL1 antisense RNA 1	644246	2,5	1,1E-05
HTATSF1P2	HIV-1 Tat specific factor 1 pseudogene 2	401233	2,5	1,3E-02
AADAC	arylacetamide deacetylase	13	2,4	8,8E-03
ALDH3B2	aldehyde dehydrogenase 3 family, member B2	222	2,4	1,9E-02
LOC100506102	uncharacterized LOC100506102	100506102	2,3	4,5E-02
ELOVL3	ELOVL fatty acid elongase 3	83401	2,2	1,2E-02
BLMH	bleomycin hydrolase	642	2,2	3,4E-02
ALOX12	arachidonate 12-lipoxygenase	239	2,2	3,7E-03
SLC46A2	solute carrier family 46, member 2	57864	2,1	1,4E-02
LY6K	lymphocyte antigen 6 complex, locus K	54742	2,1	5,7E-03
MMP3	matrix metallopeptidase 3 (stromelysin 1, progelatinase)	4314	2,1	1,3E-02
GDA	guanine deaminase	9615	2,1	1,5E-03
STK19	serine/threonine kinase 19	8859	2,0	2,0E-03
DUSP2	dual specificity phosphatase 2	1844	2,0	3,5E-03
CHI3L1	chitinase 3-like 1 (cartilage glycoprotein-39)	1116	1,9	3,8E-02
CSNK2A2	casein kinase 2, alpha prime polypeptide	1459	1,9	1,9E-02
AADACL2	arylacetamide deacetylase-like 2	344752	1,9	1,1E-02
LRMP	lymphoid-restricted membrane protein	4033	1,9	3,9E-02
CCDC113	coiled-coil domain containing 113	29070	1,9	8,1E-03
HAL	histidine ammonia-lyase	3034	1,9	1,2E-02
EREG	epiregulin	2069	1,9	1,5E-04
EGR2	early growth response 2	1959	1,8	3,3E-03
ABHD12B	abhydrolase domain containing 12B	145447	1,8	4,8E-02
SPTSSB	serine palmitoyltransferase, small subunit B	165679	1,8	1,4E-03
ID4	inhibitor of DNA binding 4, dominant negative helix-loop-helix protein	3400	1,8	3,4E-02
DHRS4-AS1	DHRS4 antisense RNA 1	55449	1,8	7,2E-04
KCNK7	potassium channel, subfamily K, member 7	10089	1,8	5,6E-03
AQP9	aquaporin 9	366	1,8	1,0E-03
APCDD1	adenomatosis polyposis coli down-regulated 1	147495	1,8	1,2E-02
CYP2E1	cytochrome P450, family 2, subfamily E, polypeptide 1	1571	1,8	1,4E-02
TCEA3	transcription elongation factor A (SII), 3	6920	1,8	3,2E-02
RBMXL1	RNA binding motif protein, X-linked-like 1	494115	1,8	8,7E-03
NUDT4	nudix (nucleoside diphosphate linked moiety X)-type motif 4	11163	1,8	1,1E-05
KCNJ2	potassium inwardly-rectifying channel, subfamily J, member 2	3759	1,8	4,2E-03
GLRX	glutaredoxin (thioltransferase)	2745	1,8	3,2E-02
RPS23	ribosomal protein S23	6228	1,8	8,6E-04
KLK9	kallikrein-related peptidase 9	284366	1,8	7,2E-03
SLC26A9	solute carrier family 26 (anion exchanger), member 9	115019	1,7	3,4E-03

RASIP1	Ras interacting protein 1	54922	1,7	4,2E-03
ACPP	acid phosphatase, prostate	55	1,7	1,8E-02
SLC5A1	solute carrier family 5 (sodium/glucose cotransporter), member 1	6523	1,7	3,3E-05
GAN	gigaxonin	8139	1,7	1,5E-02
PTPN21	protein tyrosine phosphatase, non-receptor type 21	11099	1,7	3,4E-03
SMIM5	small integral membrane protein 5	643008	1,7	1,5E-02
SLC45A4	solute carrier family 45, member 4	57210	1,7	3,9E-02
HOXB2	homeobox B2	3212	1,7	3,6E-03
EGR3	early growth response 3	1960	1,7	6,5E-03
SYTL2	synaptotagmin-like 2	54843	1,7	2,5E-02
LGALS8	lectin, galactoside-binding, soluble, 8	3964	1,7	1,0E-02
ADTRP	androgen-dependent TFPI-regulating protein	84830	1,7	3,1E-03
NA	#N/A	#N/A	1,7	3,5E-03
ELOVL7	ELOVL fatty acid elongase 7	79993	1,7	7,5E-03
RDH12	retinol dehydrogenase 12 (all-trans/9-cis/11-cis)	145226	1,6	1,3E-03
ARIH1	ariadne homolog, ubiquitin-conjugating enzyme E2 binding protein, 1 (Drosophila)	25820	1,6	7,4E-03
ZNF662	zinc finger protein 662	389114	1,6	4,2E-03
RORA	RAR-related orphan receptor A	6095	1,6	2,9E-02
PLCG2	phospholipase C, gamma 2 (phosphatidylinositol-specific)	5336	1,6	2,2E-02
ARG1	arginase 1	383	1,6	2,2E-02
PLIN2	perilipin 2	123	1,6	2,2E-02
HYAL4	hyaluronoglucosaminidase 4	23553	1,6	4,0E-02
FOXQ1	forkhead box Q1	94234	1,6	8,9E-03
ELOVL4	ELOVL fatty acid elongase 4	6785	1,6	1,5E-02
TBC1D23	TBC1 domain family, member 23	55773	1,6	3,7E-04
RORA	RAR-related orphan receptor A	6095	1,6	1,8E-02
MCUR1	mitochondrial calcium uniporter regulator 1	63933	1,6	6,8E-03
CPM	carboxypeptidase M	1368	1,6	1,1E-02
GK	glycerol kinase	2710	1,6	1,9E-02
SLC44A5	solute carrier family 44, member 5	204962	1,6	2,8E-05
GGT6	gamma-glutamyltransferase 6	124975	1,6	1,5E-02
IDE	insulin-degrading enzyme	3416	1,6	1,7E-02
NA	#N/A	#N/A	1,6	5,0E-02
DHCR24	24-dehydrocholesterol reductase	1718	1,6	1,4E-04
SNHG9	small nucleolar RNA host gene 9 (non-protein coding)	735301	1,6	3,7E-03
ELOVL1	ELOVL fatty acid elongase 1	64834	1,6	6,6E-03
BEX2	brain expressed X-linked 2	84707	1,6	1,0E-02
ZNF165	zinc finger protein 165	7718	1,6	8,8E-03
CDK5RAP2	CDK5 regulatory subunit associated protein 2	55755	1,6	4,0E-03
LAMTOR4	late endosomal/lysosomal adaptor, MAPK and MTOR activator 4	389541	1,6	8,8E-03
NPR2	natriuretic peptide receptor B/guanylate cyclase B (atrionatriuretic peptide receptor B)	4882	1,6	3,4E-02
MYRFL	myelin regulatory factor-like	196446	1,6	1,2E-02
SNTB1	syntrophin, beta 1 (dystrophin-associated protein A1, 59kDa, basic component 1)	6641	1,6	2,1E-02
ACER1	alkaline ceramidase 1	125981	1,6	7,4E-03
TGM3	transglutaminase 3	7053	1,6	4,4E-02

SLCO4A1	solute carrier organic anion transporter family, member 4A1	28231	1,5	2,7E-02
KCTD11	potassium channel tetramerization domain containing 11	147040	1,5	1,7E-02
ENDOU	endonuclease, polyU-specific	8909	1,5	1,8E-03
TNNC1	troponin C type 1 (slow)	7134	1,5	1,5E-03
HSPB8	heat shock 22kDa protein 8	26353	1,5	1,8E-02
KLK1	kallikrein 1	3816	1,5	2,9E-03
SLC30A1	solute carrier family 30 (zinc transporter), member 1	7779	1,5	4,8E-03
GPLD1	glycosylphosphatidylinositol specific phospholipase D1	2822	1,5	1,7E-03
TIMM8A	translocase of inner mitochondrial membrane 8 homolog A (yeast)	1678	1,5	3,5E-03
PLAGL1	pleiomorphic adenoma gene-like 1	5325	1,5	2,4E-03
HPSE	heparanase	10855	1,5	4,0E-02
ITGAX	integrin, alpha X (complement component 3 receptor 4 subunit)	3687	1,5	3,2E-02
KDM2B	lysine (K)-specific demethylase 2B	84678	1,5	1,7E-02
VSIG10L	V-set and immunoglobulin domain containing 10 like	147645	1,5	2,8E-02
CCSAP	centriole, cilia and spindle-associated protein	126731	1,5	3,2E-03
ATP2C2	ATPase, Ca++ transporting, type 2C, member 2	9914	1,5	3,6E-02
SLC15A1	solute carrier family 15 (oligopeptide transporter), member 1	6564	1,5	4,6E-02
ESYT3	extended synaptotagmin-like protein 3	83850	1,5	4,4E-02
MTRR	5-methyltetrahydrofolate-homocysteine methyltransferase reductase	4552	1,5	1,8E-03
PLA2G2F	phospholipase A2, group IIF	64600	1,5	1,1E-02
ZSCAN12P1	zinc finger and SCAN domain containing 12 pseudogene 1	221584	1,5	3,5E-03
OTUB2	OTU domain, ubiquitin aldehyde binding 2	78990	1,5	4,3E-02
BPIFC	BPI fold containing family C	254240	1,5	2,5E-02
PCCA	propionyl CoA carboxylase, alpha polypeptide	5095	1,5	1,1E-02
CD55	CD55 molecule, decay accelerating factor for complement (Cromer blood group)	1604	1,5	3,7E-03
HEIH	hepatocellular carcinoma up-regulated EZH2-associated long non-coding RNA	100859930	1,5	5,7E-03
HSD17B1	hydroxysteroid (17-beta) dehydrogenase 1	3292	1,5	5,1E-03
ABHD5	abhydrolase domain containing 5	51099	1,5	3,3E-02
MIR7-3HG	MIR7-3 host gene (non-protein coding)	284424	1,5	2,2E-02
PNPLA3	patatin-like phospholipase domain containing 3	80339	1,5	3,3E-03
PTGER3	prostaglandin E receptor 3 (subtype EP3)	5733	1,5	4,3E-02
CNN3	calponin 3, acidic	1266	1,5	1,9E-02
ACP6	acid phosphatase 6, lysophosphatidic	51205	1,5	3,9E-02
ABCA12	ATP-binding cassette, sub-family A (ABC1), member 12	26154	1,5	1,4E-03
ATP10B	ATPase, class V, type 10B	23120	1,5	3,6E-02
SRPK1	SRSF protein kinase 1	6732	1,5	1,8E-02
ARG2	arginase 2	384	1,5	3,5E-02
CARD18	caspase recruitment domain family, member 18	59082	1,5	2,2E-02
ZIC2	Zic family member 2	7546	1,4	1,3E-02
TREX2	three prime repair exonuclease 2	11219	1,4	2,7E-02
CYP4F22	cytochrome P450, family 4, subfamily F, polypeptide 22	126410	1,4	1,4E-02
IL22RA1	interleukin 22 receptor, alpha 1	58985	1,4	8,8E-03
EPHX3	epoxide hydrolase 3	79852	1,4	3,4E-02
PTDSS1	phosphatidylserine synthase 1	9791	1,4	1,8E-02
ANKRD9	ankyrin repeat domain 9	122416	1,4	2,2E-02
MAP3K8	mitogen-activated protein kinase kinase kinase 8	1326	1,4	2,7E-03

GLB1L2	galactosidase, beta 1-like 2	89944	1,4	2,7E-02
SCPEP1	serine carboxypeptidase 1	59342	1,4	1,4E-02
SERPINB8	serpin peptidase inhibitor, clade B (ovalbumin), member 8	5271	1,4	3,3E-02
LRRC8B	leucine rich repeat containing 8 family, member B	23507	1,4	1,9E-02
FRY	furry homolog (Drosophila)	10129	1,4	4,1E-02
KRT23	keratin 23 (histone deacetylase inducible)	25984	1,4	1,0E-02
HMOX1	heme oxygenase (decycling) 1	3162	1,4	3,1E-02
TMEM86A	transmembrane protein 86A	144110	1,4	4,0E-02
ABHD6	abhydrolase domain containing 6	57406	1,4	4,9E-02
SLC31A1	solute carrier family 31 (copper transporter), member 1	1317	1,4	2,6E-02
CERS3	ceramide synthase 3	204219	1,4	1,6E-02
SCNN1A	sodium channel, non-voltage-gated 1 alpha subunit	6337	1,4	4,5E-02
FLVCR2	feline leukemia virus subgroup C cellular receptor family, member 2	55640	1,4	1,2E-02
TM7SF2	transmembrane 7 superfamily member 2	7108	1,4	5,7E-03
GATA3	GATA binding protein 3	2625	1,4	1,5E-02
SPINK1	serine peptidase inhibitor, Kazal type 1	6690	1,4	1,4E-02
MOSPD2	motile sperm domain containing 2	158747	1,4	1,6E-02
DGAT2	diacylglycerol O-acyltransferase 2	84649	1,4	3,5E-02
OVOL1	ovo-like 1(Drosophila)	5017	1,4	3,4E-02
DEGS2	delta(4)-desaturase, sphingolipid 2	123099	1,4	2,2E-02
ASPHD2	aspartate beta-hydroxylase domain containing 2	57168	1,4	4,1E-02
NQO1	NAD(P)H dehydrogenase, quinone 1	1728	1,4	1,8E-02
SLC19A2	solute carrier family 19 (thiamine transporter), member 2	10560	1,4	3,5E-02
GGH	gamma-glutamyl hydrolase (conjugase, folylpolygammaglutamyl hydrolase)	8836	1,4	5,4E-03
SPAG1	sperm associated antigen 1	6674	1,4	2,3E-02
C12orf29	chromosome 12 open reading frame 29	91298	1,4	1,5E-03
SPRR2C	small proline-rich protein 2C (pseudogene)	6702	1,4	3,6E-02
RAPGEF4	Rap guanine nucleotide exchange factor (GEF) 4	11069	1,4	2,4E-02
GLCCI1	glucocorticoid induced transcript 1	113263	1,4	4,9E-02
SLC25A25	solute carrier family 25 (mitochondrial carrier; phosphate carrier), member 25	114789	1,4	8,5E-04
TTC39A	tetratricopeptide repeat domain 39A	22996	1,4	2,7E-02
CEBPA	CCAAT/enhancer binding protein (C/EBP), alpha	1050	1,4	2,1E-02
ETV7	ets variant 7	51513	1,4	3,7E-02
NIPAL4	NIPA-like domain containing 4	348938	1,4	3,6E-02
DIO2	deiodinase, iodothyronine, type II	1734	1,4	4,0E-02
SPTLC3	serine palmitoyltransferase, long chain base subunit 3	55304	1,4	8,8E-03
GPT2	glutamic pyruvate transaminase (alanine aminotransferase) 2	84706	1,4	3,5E-02
PET112	PET112 homolog (yeast)	5188	1,4	2,2E-02
ZRANB1	zinc finger, RAN-binding domain containing 1	54764	1,4	5,0E-02
PDZD2	PDZ domain containing 2	23037	1,4	3,5E-02
TLL2	tolloid-like 2	7093	1,4	2,7E-02
BLVRB	biliverdin reductase B (flavin reductase (NADPH))	645	1,4	1,1E-02
MID2	midline 2	11043	1,4	4,7E-02
HYMAI	hydatidiform mole associated and imprinted (non-protein coding)	57061	1,4	3,1E-02
PNPLA1	patatin-like phospholipase domain containing 1	285848	1,4	3,3E-02
FKBPL	FK506 binding protein like	63943	1,4	2,2E-02

LOC100130691	uncharacterized LOC100130691	100130691	1,4	3,1E-02
PPT2	palmitoyl-protein thioesterase 2	9374	1,4	2,7E-02
PLEKHG1	pleckstrin homology domain containing, family G (with RhoGef domain) member 1	57480	1,4	4,2E-03
DUSP1	dual specificity phosphatase 1	1843	1,4	3,4E-03
DDAH2	dimethylarginine dimethylaminohydrolase 2	23564	1,4	4,9E-02
GSTA4	glutathione S-transferase alpha 4	2941	1,4	2,2E-02
PXMP4	peroxisomal membrane protein 4, 24kDa	11264	1,4	1,1E-02
POF1B	premature ovarian failure, 1B	79983	1,4	4,1E-02
DNAJB9	DnaJ (Hsp40) homolog, subfamily B, member 9	4189	1,4	5,5E-03
RAB11FIP1	RAB11 family interacting protein 1 (class I)	80223	1,4	3,0E-02
AKIRIN1	akirin 1	79647	1,4	1,2E-02
SERPINA6	serpin peptidase inhibitor, clade A (alpha-1 antiproteinase, antitrypsin), member 6	866	1,4	3,4E-02
MGC57346	uncharacterized LOC401884	401884	1,4	1,7E-05
KLF4	Kruppel-like factor 4 (gut)	9314	1,3	3,2E-02
L2HGDH	L-2-hydroxyglutarate dehydrogenase	79944	1,3	1,2E-02
TTC39B	tetratricopeptide repeat domain 39B	158219	1,3	1,3E-02
TSPAN5	tetraspanin 5	10098	1,3	4,4E-02
FAM13C	family with sequence similarity 13, member C	220965	1,3	4,4E-02
ATG2A	autophagy related 2A	23130	1,3	4,1E-02
G6PD	glucose-6-phosphate dehydrogenase	2539	1,3	4,2E-02
HSBP1L1	heat shock factor binding protein 1-like 1	440498	1,3	1,1E-02
EGR1	early growth response 1	1958	1,3	9,4E-03
ARL5A	ADP-ribosylation factor-like 5A	26225	1,3	2,9E-02
SLC28A3	solute carrier family 28 (concentrative nucleoside transporter), member 3	64078	1,3	2,3E-02
ERC2	ELKS/RAB6-interacting/CAST family member 2	26059	1,3	3,0E-02
TEX264	testis expressed 264	51368	1,3	4,9E-02
GRHL1	grainyhead-like 1 (Drosophila)	29841	1,3	3,4E-02
CCDC58	coiled-coil domain containing 58	131076	1,3	1,6E-02
VWF	von Willebrand factor	7450	1,3	3,5E-03
AZGP1P1	alpha-2-glycoprotein 1, zinc-binding pseudogene 1	646282	1,3	4,6E-02
OVOL1	ovo-like 1(Drosophila)	5017	1,3	4,3E-02
PGRMC2	progesterone receptor membrane component 2	10424	1,3	8,8E-03
VASN	vasorin	114990	1,3	4,6E-02
EIF4A3	eukaryotic translation initiation factor 4A3	9775	1,3	3,4E-02
CARD9	caspase recruitment domain family, member 9	64170	1,3	1,2E-03
TINCR	tissue differentiation-inducing non-protein coding RNA	257000	1,3	4,7E-02
CIDEA	cell death-inducing DFFA-like effector a	1149	1,3	4,1E-02
NKPD1	NTPase, KAP family P-loop domain containing 1	284353	1,3	4,5E-02
MAST3	microtubule associated serine/threonine kinase 3	23031	1,3	4,4E-02
TSTD1	thiosulfate sulfurtransferase (rhodanese)-like domain containing 1	100131187	1,3	4,0E-03
FCHSD1	FCH and double SH3 domains 1	89848	1,3	3,7E-02
SWI5	SWI5 recombination repair homolog (yeast)	375757	1,3	1,8E-02
RALGAPA2	Ral GTPase activating protein, alpha subunit 2 (catalytic)	57186	1,3	2,1E-02
PNPLA8	patatin-like phospholipase domain containing 8	50640	1,3	5,8E-03
APOE	apolipoprotein E	348	1,3	4,1E-02
GOLGA4	golgin A4	2803	1,3	9,9E-03

BLOC1S3	biogenesis of lysosomal organelles complex-1, subunit 3	388552	1,3	6,1E-03
ARHGEF10L	Rho guanine nucleotide exchange factor (GEF) 10-like	55160	1,3	4,0E-02
PLCXD1	phosphatidylinositol-specific phospholipase C, X domain containing 1	55344	1,3	4,3E-02
TMPRSS13	transmembrane protease, serine 13	84000	1,3	4,9E-02
DNAJC1	DnaJ (Hsp40) homolog, subfamily C, member 1	64215	1,3	3,4E-02
SLC27A4	solute carrier family 27 (fatty acid transporter), member 4	10999	1,3	3,0E-02
POR	P450 (cytochrome) oxidoreductase	5447	1,3	4,2E-03
GAS7	growth arrest-specific 7	8522	1,3	3,7E-02
SYTL2	synaptotagmin-like 2	54843	1,3	1,5E-03
ANKRD22	ankyrin repeat domain 22	118932	1,3	3,6E-02
NA	#N/A	#N/A	1,3	9,5E-03
EFCAB14	EF-hand calcium binding domain 14	9813	1,3	8,8E-03
CAMP	cathelicidin antimicrobial peptide	820	1,3	1,8E-03
SEL1L	sel-1 suppressor of lin-12-like (C. elegans)	6400	1,3	2,0E-02
UGCG	UDP-glucose ceramide glucosyltransferase	7357	1,3	3,3E-02
MAPK7	mitogen-activated protein kinase 7	5598	1,3	2,3E-02
SNRK-AS1	SNRK antisense RNA 1	100873954	1,3	8,8E-03
FAM73B	family with sequence similarity 73, member B	84895	1,3	1,2E-02
USP32	ubiquitin specific peptidase 32	84669	1,3	4,3E-02
BAALC	brain and acute leukemia, cytoplasmic	79870	1,3	1,2E-03

b

Official symbol	Official full name	EntrezID	Pseudo Fc	Adjusted p-value
SPINK6	serine peptidase inhibitor, Kazal type 6	404203	-4,8	6,1E-03
DDX17	DEAD (Asp-Glu-Ala-Asp) box helicase 17	10521	-3,9	7,5E-03
CEACAM6	carcinoembryonic antigen-related cell adhesion molecule 6 (non-specific cross reacting antigen)	4680	-3,5	3,1E-02
ABHD3	abhydrolase domain containing 3	171586	-3,0	2,2E-03
POLB	polymerase (DNA directed), beta	5423	-2,7	1,7E-02
GLUL	glutamate-ammonia ligase	2752	-2,7	1,9E-02
ERP27	endoplasmic reticulum protein 27	121506	-2,4	4,6E-02
MRPL43	mitochondrial ribosomal protein L43	84545	-2,3	4,5E-02
RAB12	RAB12, member RAS oncogene family	201475	-2,2	1,8E-03
RHCG	Rh family, C glycoprotein	51458	-2,1	2,3E-02
DNAH10	dynein, axonemal, heavy chain 10	196385	-2,0	2,4E-02
LCN2	lipocalin 2	3934	-2,0	4,4E-02
TMEM200A	transmembrane protein 200A	114801	-2,0	1,8E-03
DKK1	dickkopf WNT signaling pathway inhibitor 1	22943	-1,9	2,2E-02
SRPX	sushi-repeat containing protein, X-linked	8406	-1,9	6,6E-03
HOTAIR	HOX transcript antisense RNA	100124700	-1,8	2,7E-03
PLA2G4C	phospholipase A2, group IVC (cytosolic, calcium-independent)	8605	-1,7	2,4E-02
ADAM19	ADAM metallopeptidase domain 19	8728	-1,7	4,6E-03
SPOCK1	sparc/osteonectin, cwcv and kazal-like domains proteoglycan (testican) 1	6695	-1,7	4,7E-02
KYNU	kynureninase	8942	-1,7	3,0E-02
CXCL1	chemokine (C-X-C motif) ligand 1 (melanoma growth stimulating activity, alpha)	2919	-1,7	2,7E-02

EPB41L3	erythrocyte membrane protein band 4.1-like 3	23136	-1,7	5,7E-03
GPX2	glutathione peroxidase 2 (gastrointestinal)	2877	-1,7	3,6E-02
ANXA3	annexin A3	306	-1,7	2,7E-02
ITM2A	integral membrane protein 2A	9452	-1,7	3,4E-03
C7orf10	chromosome 7 open reading frame 10	79783	-1,7	1,1E-02
IGFBP7	insulin-like growth factor binding protein 7	3490	-1,7	2,0E-02
TRIM6	tripartite motif containing 6	117854	-1,7	1,6E-02
ARPC1B	actin related protein 2/3 complex, subunit 1B, 41kDa	10095	-1,7	9,2E-04
RTTN	rotatin	25914	-1,7	5,6E-03
CD99P1	CD99 molecule pseudogene 1	401577	-1,7	1,8E-02
GLUL	glutamate-ammonia ligase	2752	-1,7	4,3E-02
GOLM1	golgi membrane protein 1	51280	-1,7	3,4E-02
CALB2	calbindin 2	794	-1,6	1,8E-02
PTHLH	parathyroid hormone-like hormone	5744	-1,6	4,4E-02
NTN4	netrin 4	59277	-1,6	1,2E-02
SPRY2	sprouty homolog 2 (Drosophila)	10253	-1,6	1,2E-02
MFAP5	microfibrillar associated protein 5	8076	-1,6	1,4E-02
C15orf65	chromosome 15 open reading frame 65	145788	-1,6	8,5E-04
ZSCAN31	zinc finger and SCAN domain containing 31	64288	-1,6	3,5E-02
VIM	vimentin	7431	-1,6	6,9E-03
HLA-B	major histocompatibility complex, class I, B	3106	-1,6	3,5E-02
HCP5	HLA complex P5 (non-protein coding)	10866	-1,6	1,7E-02
LOC100505547	uncharacterized LOC100505547	100505547	-1,6	2,5E-04
XDH	xanthine dehydrogenase	7498	-1,6	4,4E-02
FUK	fucokinase	197258	-1,6	2,7E-03
SRPX2	sushi-repeat containing protein, X-linked 2	27286	-1,5	1,3E-02
FEZ1	fasciculation and elongation protein zeta 1 (zygin I)	9638	-1,5	3,2E-02
SIDT1	SID1 transmembrane family, member 1	54847	-1,5	4,9E-02
TMPRSS11D	transmembrane protease, serine 11D	9407	-1,5	3,7E-02
ANXA1	annexin A1	301	-1,5	4,9E-02
HOXC10	homeobox C10	3226	-1,5	4,0E-03
RASSF6	Ras association (RalGDS/AF-6) domain family member 6	166824	-1,5	7,5E-03
CARD16	caspase recruitment domain family, member 16	114769	-1,5	3,3E-02
PITX1	paired-like homeodomain 1	5307	-1,5	8,1E-03
MARVELD1	MARVEL domain containing 1	83742	-1,5	2,2E-02
AFAP1L2	actin filament associated protein 1-like 2	84632	-1,5	9,8E-03
ALDH8A1	aldehyde dehydrogenase 8 family, member A1	64577	-1,5	1,9E-02
MRAP2	melanocortin 2 receptor accessory protein 2	112609	-1,5	1,8E-02
PHLDA1	pleckstrin homology-like domain, family A, member 1	22822	-1,5	3,4E-02
ARHGAP25	Rho GTPase activating protein 25	9938	-1,5	2,2E-02
EHBP1	EH domain binding protein 1	23301	-1,5	1,7E-02
SHISA2	shisa family member 2	387914	-1,5	2,3E-02
S100A6	S100 calcium binding protein A6	6277	-1,5	9,5E-03
C4orf33	chromosome 4 open reading frame 33	132321	-1,5	1,3E-02
HLA-F	major histocompatibility complex, class I, F	3134	-1,5	2,0E-02
GCHFR	GTP cyclohydrolase I feedback regulator	2644	-1,5	4,3E-02

RASSF3	Ras association (RalGDS/AF-6) domain family member 3	283349	-1,5	3,8E-02
SORL1	sortilin-related receptor, L(DLR class) A repeats containing	6653	-1,5	9,5E-03
BASP1	brain abundant, membrane attached signal protein 1	10409	-1,5	1,2E-02
SOX9	SRY (sex determining region Y)-box 9	6662	-1,5	2,2E-02
MXRA7	matrix-remodelling associated 7	439921	-1,5	4,0E-02
FNDC3B	fibronectin type III domain containing 3B	64778	-1,5	2,2E-02
EHBP1	EH domain binding protein 1	23301	-1,5	2,3E-02
KIAA1598	KIAA1598	57698	-1,5	2,5E-04
HMGA2	high mobility group AT-hook 2	8091	-1,5	1,7E-02
GSTO1	glutathione S-transferase omega 1	9446	-1,5	2,5E-02
RAC2	ras-related C3 botulinum toxin substrate 2 (rho family, small GTP binding protein Rac2)	5880	-1,5	2,9E-02
C4orf48	chromosome 4 open reading frame 48	401115	-1,5	3,2E-02
ANKRD36BP2	ankyrin repeat domain 36B pseudogene 2	645784	-1,5	5,7E-03
IAH1	isoamyl acetate-hydrolyzing esterase 1 homolog (S. cerevisiae)	285148	-1,5	1,5E-02
ITGB6	integrin, beta 6	3694	-1,5	3,2E-02
MFAP2	microfibrillar-associated protein 2	4237	-1,4	2,9E-02
BACE2	beta-site APP-cleaving enzyme 2	25825	-1,4	4,0E-02
PLEKHH2	pleckstrin homology domain containing, family H (with MyTH4 domain) member 2	130271	-1,4	4,2E-03
ACTB	actin, beta	60	-1,4	1,4E-03
TRIM2	tripartite motif containing 2	23321	-1,4	3,2E-02
YWHAH	tyrosine 3-monooxygenase/tryptophan 5-monooxygenase activation protein, eta polypeptide	7533	-1,4	2,2E-02
FXYD5	FXYD domain containing ion transport regulator 5	53827	-1,4	4,1E-02
SLC12A8	solute carrier family 12, member 8	84561	-1,4	1,1E-02
RHOBTB1	Rho-related BTB domain containing 1	9886	-1,4	1,1E-02
ARL6IP5	ADP-ribosylation-like factor 6 interacting protein 5	10550	-1,4	4,7E-02
ENC1	ectodermal-neural cortex 1 (with BTB domain)	8507	-1,4	4,6E-02
TACC1	transforming, acidic coiled-coil containing protein 1	6867	-1,4	2,2E-02
GALNT7	UDP-N-acetyl-alpha-D-galactosamine:polypeptide N-acetylgalactosaminyltransferase 7 (GalNAc-T7)	51809	-1,4	1,2E-02
NT5E	5'-nucleotidase, ecto (CD73)	4907	-1,4	1,5E-02
PLAU	plasminogen activator, urokinase	5328	-1,4	1,1E-02
CDH3	cadherin 3, type 1, P-cadherin (placental)	1001	-1,4	4,0E-03
KRT17	keratin 17	3872	-1,4	3,0E-02
BLVRA	biliverdin reductase A	644	-1,4	5,7E-03
ACOT9	acyl-CoA thioesterase 9	23597	-1,4	2,9E-02
SDCBP	syndecan binding protein (syntenin)	6386	-1,4	5,1E-03
PEX6	peroxisomal biogenesis factor 6	5190	-1,4	2,2E-02
SMURF2	SMAD specific E3 ubiquitin protein ligase 2	64750	-1,4	2,4E-02
ENC1	ectodermal-neural cortex 1 (with BTB domain)	8507	-1,4	3,5E-02
FAM114A1	family with sequence similarity 114, member A1	92689	-1,4	2,3E-02
ARHGAP18	Rho GTPase activating protein 18	93663	-1,4	2,0E-02
PMAIP1	phorbol-12-myristate-13-acetate-induced protein 1	5366	-1,4	4,0E-03
ARL14	ADP-ribosylation factor-like 14	80117	-1,4	3,2E-02
ENTPD3	ectonucleoside triphosphate diphosphohydrolase 3	956	-1,4	4,7E-02
LIMA1	LIM domain and actin binding 1	51474	-1,4	3,7E-03
EMX2	empty spiracles homeobox 2	2018	-1,4	2,2E-02

CIB1	calcium and integrin binding 1 (calmyrin)	10519	-1,4	1,5E-02
B4GALT5	UDP-Gal:betaGlcNAc beta 1,4- galactosyltransferase, polypeptide 5	9334	-1,4	4,2E-03
TTLL7	tubulin tyrosine ligase-like family, member 7	79739	-1,4	1,9E-02
LAMB1	laminin, beta 1	3912	-1,4	9,1E-03
C4orf19	chromosome 4 open reading frame 19	55286	-1,4	4,1E-02
FOXP1-IT1	FOXP1 intronic transcript 1 (non-protein coding)	100506815	-1,4	3,4E-02
DSG2	desmoglein 2	1829	-1,4	4,6E-02
ALDH7A1	aldehyde dehydrogenase 7 family, member A1	501	-1,4	2,7E-02
PSTPIP2	proline-serine-threonine phosphatase interacting protein 2	9050	-1,4	1,4E-02
CASP7	caspase 7, apoptosis-related cysteine peptidase	840	-1,4	3,5E-03
TUBGCP4	tubulin, gamma complex associated protein 4	27229	-1,4	3,6E-03
OCIAD2	OCIA domain containing 2	132299	-1,4	5,7E-03
UAP1	UDP-N-acteylglucosamine pyrophosphorylase 1	6675	-1,4	1,8E-02
FEZ2	fasciculation and elongation protein zeta 2 (zygin II)	9637	-1,4	1,7E-02
KCTD3	potassium channel tetramerization domain containing 3	51133	-1,4	1,9E-02
RICTOR	RPTOR independent companion of MTOR, complex 2	253260	-1,4	4,9E-02
CSNK1D	casein kinase 1, delta	1453	-1,4	2,0E-02
PALLD	palladin, cytoskeletal associated protein	23022	-1,4	1,6E-02
ARSJ	arylsulfatase family, member J	79642	-1,4	2,4E-03
CCDC109B	coiled-coil domain containing 109B	55013	-1,4	1,4E-03
IFI16	interferon, gamma-inducible protein 16	3428	-1,4	1,2E-02
GLCE	glucuronic acid epimerase	26035	-1,4	1,9E-02
ARHGEF40	Rho guanine nucleotide exchange factor (GEF) 40	55701	-1,4	2,6E-02
ZNF816	zinc finger protein 816	125893	-1,4	2,4E-02
CKAP2	cytoskeleton associated protein 2	26586	-1,4	3,6E-02
AKR1B1	aldo-keto reductase family 1, member B1 (aldose reductase)	231	-1,4	4,0E-02
FCHSD2	FCH and double SH3 domains 2	9873	-1,3	7,0E-03
IGF1R	insulin-like growth factor 1 receptor	3480	-1,3	4,7E-02
EOGT	EGF domain-specific O-linked N-acetylglucosamine (GlcNAc) transferase	285203	-1,3	4,0E-02
C1orf74	chromosome 1 open reading frame 74	148304	-1,3	4,0E-02
PRKACB	protein kinase, cAMP-dependent, catalytic, beta	5567	-1,3	1,4E-02
CYSTM1	cysteine-rich transmembrane module containing 1	84418	-1,3	2,1E-02
ETHE1	ethylmalonic encephalopathy 1	23474	-1,3	2,4E-02
SERPINF1	serpin peptidase inhibitor, clade F (alpha-2 antiplasmin, pigment epithelium derived factor), member 1	5176	-1,3	4,5E-02
BOLA3	bolA family member 3	388962	-1,3	1,7E-02
CST3	cystatin C	1471	-1,3	3,1E-02
ST6GALNAC2	ST6 (alpha-N-acetyl-neuraminyl-2,3-beta-galactosyl-1,3)-N-acetylgalactosaminide alpha-2,6-sialyltransferase 2	10610	-1,3	4,1E-02
DDX17	DEAD (Asp-Glu-Ala-Asp) box helicase 17	10521	-1,3	2,5E-02
RNF19B	ring finger protein 19B	127544	-1,3	1,6E-02
LPGAT1	lysophosphatidylglycerol acyltransferase 1	9926	-1,3	4,6E-03
LINC00467	long intergenic non-protein coding RNA 467	84791	-1,3	9,3E-03
MYOF	myoferlin	26509	-1,3	4,2E-03
PHYH	phytanoyl-CoA 2-hydroxylase	5264	-1,3	4,5E-02
FAM213A	family with sequence similarity 213, member A	84293	-1,3	4,8E-03
RHOC	ras homolog family member C	389	-1,3	9,4E-03
LOC100506870	uncharacterized LOC100506870	100506870	-1,3	4,4E-02

CAPNS1	calpain, small subunit 1	826	-1,3	2,7E-02
UBN1	ubinuclein 1	29855	-1,3	1,6E-02
SNX7	sorting nexin 7	51375	-1,3	2,7E-02
EDN1	endothelin 1	1906	-1,3	2,5E-02
DOCK11	dedicator of cytokinesis 11	139818	-1,3	1,4E-02
IQGAP1	IQ motif containing GTPase activating protein 1	8826	-1,3	4,4E-02
SHISA5	shisa family member 5	51246	-1,3	3,2E-02
PFN1	profilin 1	5216	-1,3	3,5E-03
PRDX3	peroxiredoxin 3	10935	-1,3	3,6E-02
RNPEP	arginyl aminopeptidase (aminopeptidase B)	6051	-1,3	2,2E-02
SH3RF1	SH3 domain containing ring finger 1	57630	-1,3	1,5E-02
ARHGAP5-AS1	ARHGAP5 antisense RNA 1 (head to head)	84837	-1,3	2,1E-02
TRIM38	tripartite motif containing 38	10475	-1,3	8,2E-03
GALNT2	UDP-N-acetyl-alpha-D-galactosamine:polypeptide N-acetylgalactosaminyltransferase 2 (GalNAc-T2)	2590	-1,3	3,4E-02
DFNA5	deafness, autosomal dominant 5	1687	-1,3	9,5E-03
FN3KRP	fructosamine 3 kinase related protein	79672	-1,3	4,9E-02
GALNT14	UDP-N-acetyl-alpha-D-galactosamine:polypeptide N-acetylgalactosaminyltransferase 14 (GalNAc-T14)	79623	-1,3	3,6E-02
TMEM14A	transmembrane protein 14A	28978	-1,3	2,1E-02
CD59	CD59 molecule, complement regulatory protein	966	-1,3	3,2E-02
PHC2	polyhomeotic homolog 2 (Drosophila)	1912	-1,3	2,2E-03
HLA-J	major histocompatibility complex, class I, J (pseudogene)	3137	-1,3	4,6E-02
CDC27	cell division cycle 27	996	-1,3	4,9E-02
ACER3	alkaline ceramidase 3	55331	-1,3	3,2E-02

Supplementary Table S1. List of regulated genes between epidermis from RS with cells of Caucasian skin type when compared to those with cells of African skin type. Genes up-regulated (**a**), genes down-regulated (**b**) identified by micro-array analysis with Fc ≥ 1.3 and FDR adjusted $P \leq 0.05$.

a

Related metabolism	Official symbol	Function	Official full name	Pseudo Fc	Adjusted p-value
Fatty acyls/fatty acid	FAR2	enzyme	fatty acyl CoA reductase 2	2.6	0.010
	AADAC	enzyme	arylacetamide deacetylase	2.4	0.008
	ALOX12	enzyme	arachidonate 12-lipoxygenase	2.2	0.004
	ELOVL3	enzyme	ELOVL fatty acid elongase 3	2.2	0.012
	AADACL2	enzyme	arylacetamide deacetylase-like 2	1.9	0.011
	ELOVL7	enzyme	ELOVL fatty acid elongase 7	1.7	0.008
	ELOVL4	enzyme	ELOVL fatty acid elongase 4	1.6	0.015
	ELOVL1	enzyme	ELOVL fatty acid elongase 1	1.6	0.007
Phospholipid	SLC44A5	transporter	solute carrier family 44, member 5	5.7	0.00001
	ABHD3	enzyme	abhydrolase domain-containing protein 3	-3.0	0.002
Sphingolipid/ceramid	SPTSSB	enzyme	serine palmitoyltransferase, small subunit B	1.8	0.001
	ACER1	enzyme	alkaline ceramidase 1	1.6	0.007
	DEGS2	enzyme	delta(4)-desaturase, sphingolipid	1.4	0.022
	CERS3	enzyme	ceramide synthase 3	1.4	0.016
	SPTLC3	enzyme	serine palmitoyltransferase, long chain base subunit 3	1.4	0.009
	ACER3	enzyme	Alkaline Ceramidase 3	-1.3	0.032
	GLA	enzyme	galactosidase, alpha	1.3	0.017
	UGCG	enzyme	UDP-glucose ceramide glucosyltransferase	1.3	0.033
	ABCA12	transporter	ATP-binding cassette, sub-family A (ABC1), member 12	1.5	0.001
Sterol lipid/ cholesterol	CH25H	enzyme	cholesterol 25-hydroxylase	2.9	0.013
	DHCR24	enzyme	24-dehydrocholesterol reductase	1.6	0.0001
Glycerolipid	GK	enzyme	glycerol kinase	1.6	0.019
	PNPLA3	enzyme	Patatin-like phospholipase domain containing 3	1.5	0.003
	DGAT2	enzyme	Diacylglycerol O-acyltransferase 2	1.4	0.035
	ALDH7A1	enzyme	Aldehyde dehydrogenase 7 family, member A1	-1.4	0.027
	AKR1B1	enzyme	aldo-keto reductase family 1, member B1	-1.4	0.04
Miscellaneous lipid related genes	PLIN2	binding	perilipin 2	1.6	0.022
	PNPLA1	enzyme	patatin-like phospholipase domain containing 1	1.4	0.033

b

Related metabolism	Official symbol	Function	Official full name	Pseudo Fc	Adjusted p value
Filaggrin processing	BLMH	enzyme	bleomycin hydrolase	2.2	0.034
	HAL	enzyme	histidine ammonia-lyase	1.9	0.012
	ARG1	enzyme	arginase-1	1.6	0.02
Proteases/inhibitors	SPINK6	inhibitor	serine peptidase inhibitor, Kazal type	-4.8	0.006
	KLK1	enzyme	kallikrein 1	1.5	0.003
	KLK9	enzyme	kallikrein 9	1.8	0.007
	KLK5	enzyme	kallikrein 5	1.3	0.053
	SERPINB3	inhibitor	serpin peptidase inhibitor, clade B (ovalbumin), member	1.3	0.05
Cross-linking	TGM3	enzyme	transglutaminase 3	1.6	0.044
Transcription factor	POU2F3		POU class 2 homeobox 3	1.3	0.010
Calcium influx	PTH2R	receptor	parathyroid hormone 2 receptor	2.8	0.004

Supplementary Table S2. Tables of regulated genes identified by transcriptomic analysis related to lipid metabolism (**a**) and differentiation (**b**) processes in the epidermis of Caucasian RS models when compared to the epidermis of African RS models.

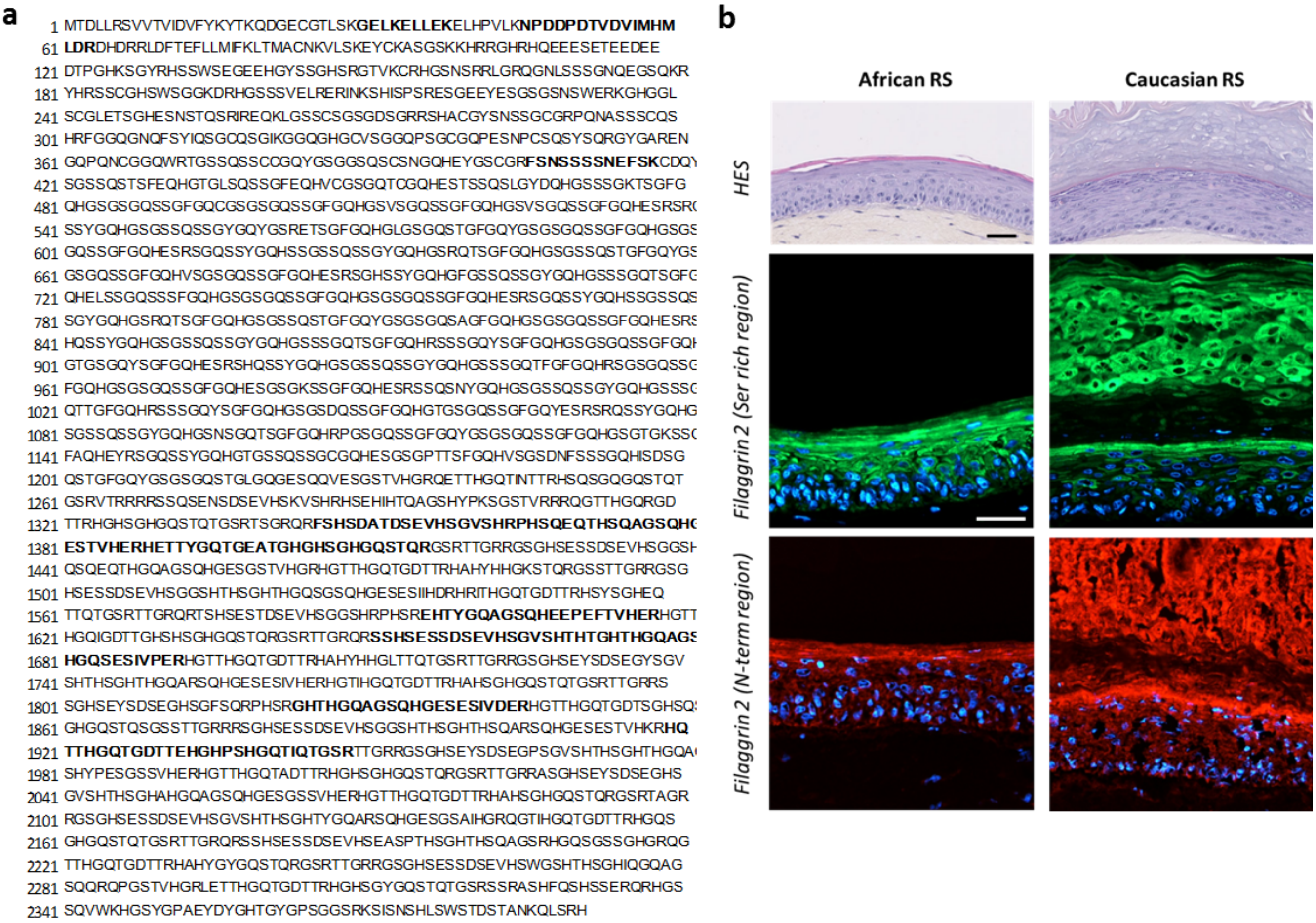

Supplementary Figure S1. (a) Identified peptides (in bold) within the filaggrin 2 protein sequence and which were differentially regulated in the epidermis from African A1 and Caucasian C2 RS. Note that peptides have been found in the N terminal, internal and the C terminal domains of filaggrin 2 sequence (**b**) Histology, expression patterns of Ser-rich (green) and N-terminal (red) regions for filaggrin 2 in African A1 and Caucasian C2 *in vitro* epidermis. Nuclei were counterstained with DAPI. Scale bars: 50 µm.

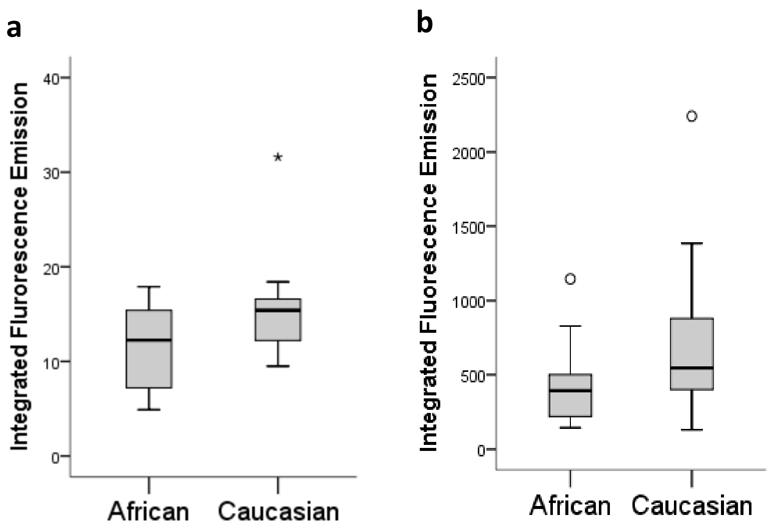

Supplementary Figure S2. (a) Quantification of fluorescent detection for Loricrin (a) and Involucrin (**b**) proteins in epidermis of African and Caucasian human skin (n=10 per skin type).

Detailed Methods

African skin type		Caucasian skin type	
Donor	Age	Donor	Age
A1	23	C1	25
A2	22	C2	22
A3	26	C3	25
A4	27	C4	23
A5	18	C5	19
A6	25	C6	19
A7	29	C7	21
A8	30	C8	26
A9	30	C9	29
A10	39	C10	37
A11	40	C11	37
A12	42	C12	42

Supplementary Table S3. List of skin samples used in this report. Human skin samples were obtained from 18 to 42 years-old female from breast surgery. African and Caucasian skin types were classified as phototype II/III/IV and phototype V/VI. Keratinocytes and papillary fibroblasts have been isolated from skin samples from donors A1 to A4 and C1 to C4.

Gene expression analysis using microarrays

Overall gene expression of epidermis from RS was performed for four donors per skin type. Each condition was prepared in triplicate experiments. Epidermis was manually separated from dermis and rinsed with buffer before the extraction.

Sample processing was performed at an Affymetrix Service Provider and Core Facility, "KFB - Center of Excellence for Fluorescent Bioanalytics" (Regensburg, Germany; www.kfb-regensburg.de).

Total RNA was extracted according to the "Purification of total RNA from animal and human cells" protocol of the RNeasy Mini Kit (Qiagen, Hilden, Germany). In brief, the samples were homogenised in 800 µl RLT buffer using Precellys CK14 ceramic beads (Bertin technology, Montigny-le-Bretonneux, France) for 2 cycles of 20 seconds at 6500 rpm. Samples were centrifuged at 12,000 x g for 3 minutes. One volume of 70 % ethanol was added to the removed supernatant and the mixture was applied to RNeasy Mini spin columns, followed by an on-column DNase digestion and several wash steps. Finally, total RNA was eluted in 30 µl of nuclease free water. Purity and integrity of the RNA was assessed on the Agilent 2100 Bioanalyzer with the RNA 6000 Nano LabChip reagent set (Agilent Techn., Palo Alto, CA, USA).

Sample preparation for microarray hybridization was carried out as described in the Affymetrix GeneChip 3' IVT Express Kit User Manual (Affymetrix Inc., Santa Clara, CA, USA). In brief, 250 ng of total RNA were reverse transcribed into double-stranded copy DNA (cDNA), followed by an *in vitro* transcription generating biotin-labelled amplified RNA (aRNA). The length of the purified aRNA products was assessed using an Agilent 2100 bioanalyzer (Agilent Technologies, Palo Alto, USA). Following fragmentation, 6 µg aRNA were hybridized on Affymetrix Human Genome U133+ PM Array Plates. For hybridization, washing, staining and scanning, the Affymetrix GeneTitan system, controlled by the Affymetrix GeneChip Command Console software v4.2, was used.

Microarray data analyses were performed by AltraBio society (Lyon, France; www.altrabio.com). The Affymetrix CEL files were analysed using the opensource software R with the Bioconductor suite of statistical packages. Firstly, Raw probe signals were treated with the Robust Multi-chip Analysis (RMA) (Irizarri *et al.*, 2003), consisting on the three following steps: Background Adjustment, Quantile Normalization, and Probe Signal Summarization. For each probe set of each sample, the probe signals were summarised into a

single value using a modified version of release 17.1 of the Entrez-Gene based CDF from The Michigan Center for Biological Information. Then, non-informative genes were filtered using the I/NI algorithm (Talloen W *et al.*, 2007). Mixed linear regression models (Pinheiro J *et al.*, 2013) were applied to identify differentially expressed genes between the different models with a random 'Donor' effect to account for the hierarchical nature of the experimental design. The empirical Bayes method was used to compute moderated *P*-values that were then corrected for multiple comparisons using the Benjamini and Hochberg's false discovery rate (FDR) controlling procedure.

Gene set enrichment analyses based on the hypergeometric distribution applied to the Gene Ontology (Ashburner M et al., 2000) and review of the PubMed database were performed to characterize the differentially expressed genes.

Quantitative proteomic analysis

Stable isotope labelling by amino acids in cell culture (SILAC) was performed to detect differences in synthetized protein amount from the epidermis between African and Caucasian RS models (donor A1 *vs* donor C2). RS were produced in triplicate experiments per donor. Specifically, RS were cultured in MEM deficient in lysine and arginine (Gibco/ThermoFischer Sc., Waltham MA, USA) supplemented with "heavy" L-Lysine-2HCL, 13C6, 15N and L-Arginine-HCL, 13C6 (ThermoFischer Sc.) for Caucasian skin models or "light" L-Lysine-2HCL and L-Arginine-HCL (ThermoFischer Sc.) for African skin models, NEA, sodium pyruvate, penicillin-streptomycin, amphotericin B, EGF, cholera toxin, hydrocortisone and 10% dialyzed FCS (ThermoFischer Sc.). Proteins were extracted from epidermis after 9 days of emersion phase, with buffer Tris 50mM, 0.3% empigen and protease inhibitors. Three combinations of proteins from Caucasian and African RS (heavy and light) were analysed together by mass spectrometry. Pooled proteins were digested and fractioned

by reverse-phase HPLC at high pH. Fractions were analysed by LC-MS/MS (5600+ triple TOF, AB Sciex, Ontario, Canada) and each pair of identical peptides was discriminated according to the difference in mass. The ratio of intensity for the paired identical peptides in mass spectra of each pool reflected the difference in amount for each protein between the two conditions. Identification and quantification of proteins were performed using Mascot (Matrix Science) and ProteinPilot 4.5 (AB Sciex). The list of identified proteins was supported with the combined results Mascot and statistics Scaffold tools. Proteins identified by at least 2 peptides or more and with 1% false discovery rate (FDR) were selected and considered to be differentially produced between epidermis of African and Caucasian skin models when differences were observed in a minimum of 2/3 experiments and a ratio with a P value <0.05.

Talloen, W. *et al.* I/NI-calls for the exclusion of non-informative genes: a highly effective filtering tool for microarray data. *Bioinformatics* **23,** 2897-2902 (2007).

Official gene symbol	Probe
SLC44A5	Hs01120485_m1
DHCR24	Hs00207388_m1
CERS3	Hs00698859_m1
EVOLV3	Hs00537016_m1
FAR2	Hs00216461_m1
ACER1	Hs00370322_m1
ABHD3	Hs00370641_m1
POU2F3	Hs00205009_m1
BLMH	Hs00166071_m1
TGM3	Hs00162752_m1
SPINK6	Hs02577364_m1
KRT14	Hs00265033_m1
FLG	Hs00856927_g1
FLG2	Hs00418578_m1
GUSB	Hs99999908_m1
TBP	Hs00427620_m1
YWHAZ	Hs03044281_g1

Supplementary Table S4. TaqMan gene expression assays used for real time PCR
